# Anti-tick vaccine candidate subolesin is important for blood feeding and innate immune gene expression in soft ticks

**DOI:** 10.1371/journal.pntd.0011719

**Published:** 2023-11-07

**Authors:** Krittika Nandy, Comfort Tamakloe, Daniel E. Sonenshine, Hameeda Sultana, Girish Neelakanta

**Affiliations:** 1 Department of Biomedical and Diagnostic Sciences, College of Veterinary Medicine, University of Tennessee, Knoxville, Tennessee, United States of America; 2 Department of Biological Sciences, Old Dominion University, Norfolk, Virginia, United States of America; 3 The University of Queensland- Ochsner Clinical School, Jefferson, Loiusiana, United States of America; 4 Vector Molecular Biology Section, Laboratory of Malaria and Vector Research, National Institute of Allergy and Infectious Diseases, National Institutes of Health, Rockville, Maryland, United States of America; National Institutes of Health, UNITED STATES

## Abstract

Subolesin is a conserved molecule in both hard and soft ticks and is considered as an effective candidate molecule for the development of anti-tick vaccine. Previous studies have reported the role of subolesin in blood feeding, reproduction, development, and gene expression in hard ticks. However, studies addressing the role of subolesin in soft ticks are limited. In this study, we report that subolesin is not only important in soft tick *Ornithodoros turicata americanus* blood feeding but also in the regulation of innate immune gene expression in these ticks. We identified and characterized several putative innate immune genes including Toll, Lysozyme precursor (Lp), fibrinogen-domain containing protein (FDP), cystatin and ML-domain containing protein (MLD) in *O*. *turicata americanus* ticks. Quantitative real-time polymerase chain reaction analysis revealed the expression of these genes in both *O*. *turicata americanus* salivary glands and midgut and in all developmental stages of these soft ticks. Significantly increased expression of *fdp* was noted in salivary glands and midgut upon *O*. *turicata americanus* blood feeding. Furthermore, RNAi-mediated knockdown of *O*. *turicata americanus* subolesin expression affected blood feeding and innate immune gene expression in these ticks. Significant downregulation of *toll*, *lp*, *fdp*, *cystatin*, and *mld* transcripts was evident in *sub*-dsRNA-treated ticks when compared to the levels noted in mock-dsRNA-treated control. Collectively, our study not only reports identification and characterization of various innate immune genes in *O*. *turicata americanus* ticks but also provides evidence on the role of subolesin in blood feeding and innate immune gene expression in these medically important ticks.

## Author summary

Soft ticks belonging to *Ornithodoros* genus such as *O*. *turicata* are important vectors for human relapsing fever agent. Tick-borne relapsing fever is a neglected disease. Therefore, studies that address molecular aspects of the vector biology of these soft ticks are important. In this study, we identified and partially sequenced several innate immune genes in *O*. *turicata americanus* ticks. Studies with hard ticks have shown that subolesin plays an important role in tick feeding, reproduction, and regulation of gene expression. However, studies deciphering the role of subolesin in soft ticks are limited. In this study, we report that *O*. *turicata americanus* subolesin not only plays a role in blood feeding but also regulates innate immune gene expression in these ticks.

## Introduction

Ticks are obligate and hematophagous parasitic arthropods that serve as major vectors for several human and animal pathogens [1]. They transmit pathogens to the vertebrate host during the blood meal [2]. There are three families of ticks in nature: a) Argasidae (soft ticks) B) Ixodidae (hard ticks) and C) Nuttalliellidae [3]. Unlike hard ticks, soft ticks lack a hard scutum on their body and their hypostome/capitulum/mouth parts are not readily visible [4]. The integument in soft ticks is pliable and rough-textured [5]. In addition, soft ticks differ from hard ticks by feeding rapidly, usually in about 45 min to 2 h and feed multiple times [6]. The life stages of soft ticks include eggs, larvae, up to seven nymphal instars and adults [6,7]. After every feeding and mating, a female tick could lay several batches of eggs [6,8]. Humans are incidental hosts for these argasid ticks [9]. Strategies to eradicate soft ticks and other ticks basically involves the use of acaricides that was noted to be ineffective in many cases [10]. Therefore, studies addressing the development of new strategies to target these, and other medically relevant ticks are important.

Recent studies highlight that anti-tick vaccines may be considered among the most effective and safest approach to control ticks and tick-borne pathogens [11–15]. In this regard, the tick protective antigenic molecule, subolesin, has been identified as a potential vaccine candidate for curbing tick infestations and pathogen transmission and infections in hard ticks [11–13,16–18]. Subolesin is an evolutionarily conserved protein that plays significant roles in tick blood feeding, development, reproduction, and tick innate immunity in hard ticks [16,18–20]. Immunization studies with recombinant subolesin protein revealed significantly reduced tick infestations on animals [21]. Furthermore, knockdown of subolesin affected blood feeding and reproduction in hard ticks [17,19,20]. A more recent study demonstrates that vaccination with recombinant subolesin-based vaccines provides cross-tick species protection [22]. These studies emphasize the importance of the development of subolesin-based strategies to target multiple ticks.

Soft ticks belonging to *Ornithodoros* species are important vectors for human relapsing fever, African swine fever and canine jaundice agents [23–26]. In the United States, *O*. *hermsii* and *O*. *turicata* are the primary vectors for the transmission of human relapsing fever agents [23,26]. Subolesin orthologs have been identified in more than 15 tick species including *Ornithodoros* species [16,19,27,28]. Vaccination studies with subolesin recombinant proteins from *O*. *moubata* and *O*. *erraticus* partially impaired oviposition in these ticks [27]. A study from hard ticks noted that silencing of subolesin affected expression of genes involved in anticoagulation, innate immunity, chromatin structure and other pathways [20]. The evidence of the role of subolesin in different arthropod physiological processes are basically noted from studies carried out with hard ticks [16,18–20]. However, the role for subolesin in various physiological processes in soft ticks needs to be explored. In this study, we not only identified and characterized several innate immune genes in soft ticks *O*. *turicata americanus* but also noted that subolesin is important for blood feeding and innate immune gene expression in these ticks.

## Results

### Identification and sequencing of *Ornithodoros turicata americanus* orthologs of cystatin, fibrinogen domain-containing protein (FDP) and ML (MD-2 related lipid-recognition)-domain containing protein (MLD) orthologs

**Fig 1:**
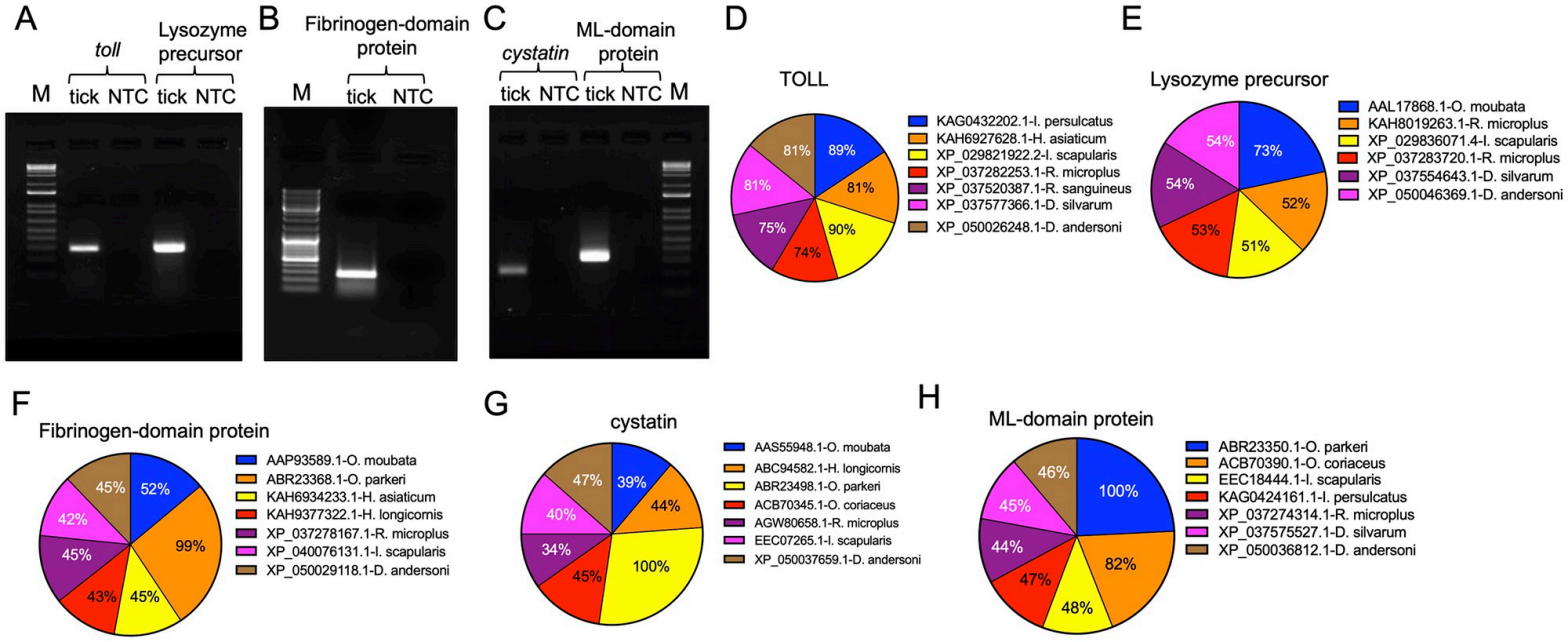
PCR amplification of *O*. *turicata americanus* innate immune gene fragments and bioinformatic analysis. **A)** Agarose gel image showing the PCR products from initial amplification of *O*. *turicata americanus toll* and *lp* (A), *fdp* (B), *cystatin* and *mld* (C) gene fragments from unfed adult female ticks. M indicates the marker and NTC indicates no template control. Pie charts showing percent identity of *O*. *turicata americanus* TOLL (D), Lp (E), FDP (F), cystatin (G) and MLD (H) proteins with orthologs from other ticks is shown. Percent identity was determined using MegAlign software in DNASTAR.

Orthologs of Toll, cystatin, fibrinogen domain-containing protein (FDP), Lysozyme precursor (Lp) and ML-domain proteins (MLD) have been identified in several ticks [14,29–35]. To identify whether *O*. *turicata americanus* also encode these genes in its genome, we first screened the previously published transcriptome data [36]. Analysis of the RNA sequences from the transcriptomic study revealed the presence of orthologs of Toll, lysozyme-precursor and MLD genes in *O*. *turicata americanus*. Oligonucleotides were designed based on these GenBank deposited *O*. *turicata americanus toll*, *lp*, or *mld* genes and *O*. *parkeri cystatin* and *fdp* gene sequences (S1 Table). QRT-PCR analysis confirmed expression of these five genes in unfed *O*. *turicata americanus* female ticks (Fig 1A). Annotation of these GenBank deposited transcriptomic data revealed presence of an early stop codon in *O*. *turicata americanus* MLD amino acid sequence. Therefore, PCR was performed to amplify larger fragments of *O*. *turicata americanus mld* (S1 Fig). In addition, larger PCR fragments of *fdp* and *cystatin* genes were amplified (S1 Fig). The PCR fragments were cloned in to pGEMT vector and three clones for each gene were sequenced. Alignment of nucleotide (S2–S4 Figs) and amino acid (S5 Fig) sequences of 3 clones of *O*. *turicata americanus fdp* (S2 Fig), *cystatin* (S3 Fig) and *mld* (S4 Fig) revealed no changes in their respective sequences. The nucleotide sequence of *O*. *turicata americanus* clone 1 of each gene was deposited in GenBank. The following are the *O*. *turicata americanus* accession numbers: *fdp* (OQ330853), *cystatin* (OQ330852), and *mld* (OQ330854).

### Comparison of *O*. *turicata americanus* Toll, lysozyme precursor, FDP, cystatin, and MLD with orthologs from hard and soft ticks

The annotated amino acid sequences of O. *turicata americanus* Toll, Lp, FDP, cystatin, and MLD were aligned with their counterparts from other ticks. Alignment of *O*. *turicata americanus* Toll revealed approximately 81% identity with *Hyalomma asiaticum*, *Dermacentor silvarum* and *D*. *andersoni* Toll orthologs. In addition, *O*. *turicata americanus* Toll shares 89–90% identity with *Ixodes persulcatus* and *I*. *scapularis* and 74–75% identity with *Rhipicephalus microplus* and *Rhipicephalus sanguineus* Toll orthologs (Figs 1D and S6). Alignment of *O*. *turicata americanus* lysozyme precursor (Lp) revealed approximately 51–54% identity with *R*. *microplus*, *I*. *scapularis*, *D*. *silvarum* and *D*. *andersoni* Lp orthologs (Figs 1E and S6). However, *O*. *turicata americanus* Lp showed higher percentage (73%) of identity with *Ornithodoros moubata* Lp ortholog (Figs 1E and S6). Alignment of *O*. *turicata americanus* FDP showed approximately 42–45% identity with *H*. *asiaticum*, *Haemaphysalis longicornis*, *R*. *microplus*, *I*. *scapularis* and *D*. *andersoni* FDP orthologs (Figs 1F and S6). In addition, *O*. *turicata americanus* FDP showed approximately 52% and 99% identity with *O*. *moubata* and *Ornithodoros parkeri* FDP orthologs, respectively (Figs 1F and S6). Alignment of *O*. *turicata americanus* cystatin showed approximately 34–47% identity with *O*. *moubata*, *H*. *longicornis*, *Ornithodoros coriaceus*, *R*. *microplus*, *I*. *scapularis*, *D*. *andersoni* and 100% identity with *O*. *parkeri* cystatin orthologs (Figs 1G and S7). Alignment of *O*. *turicata americanus* MLD amino acid sequences showed 45–48% identity with *I*. *scapularis*, *I*. *persulcatus*, *R*. *microplus*, *D*. *silvarum and D*. *andersoni* MLD orthologs (Figs 1H and S7). In addition, *O*. *turicata americanus* MLD amino acid sequence showed higher percentage (82 and 100%) of identity with *O*. *coriaceus* and *O*. *parkeri* MLD orthologs, respectively (Figs 1H and S7).

**Fig 2:**
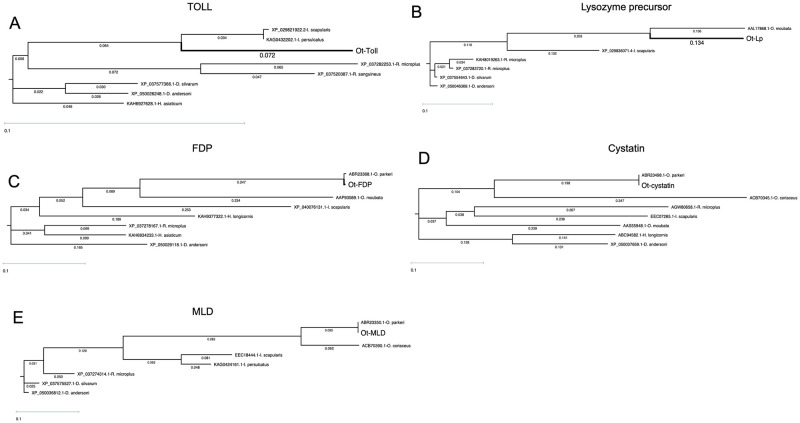
Sequence analyses of *O*. *turicata americanus* innate immune genes. Phylogenetic tree analysis for *O*. *turicata americanus* TOLL (A), Lp (B), FDP (C), cystatin (D) and MLD (E) with orthologs from other hard and soft ticks is shown. All sequences were aligned using DNASTAR MegAlign software and phylogenetic trees were generated using BIONJ algorithm and Neighbor-Joining (BIONJ) method.

Furthermore, phylogenetic analysis revealed that *O*. *turicata americanus* Toll shared the same clade with *I*. *scapularis* and *I*. *persulcatus* Toll (Fig 2A). Toll amino acid sequences from *Rhipicephalus* and *Dermacentor* species formed two different clades far from the clade shared by *O*. *turicata americanus* and *Ixodes* species (Fig 2A). In addition, Toll sequence from *H*. *asiaticum* formed a different clade away from all the analyzed Toll sequences. The phylogenetic analysis of Lp revealed that amino acid sequences of *O*. *turicata americanus* and *O*. *moubata* Lp fall within the same clade close to *I*. *scapularis* sequence (Fig 2B). However, the other analyzed Lp formed different clades away from *O*. *turicata americanus*, *O*. *moubata* and *I*. *scapularis* Lp clade (Fig 2B). The phylogenetic analysis of FDP revealed that *O*. *turicata americanus* and *O*. *parkeri* FDPs fall within the same clade close to *O*. *moubata* FDP (Fig 2C). FDPs from *I*. *scapularis* and *H*. *longicornis* were close to the clade formed by *Ornithodoros* species (Fig 2C). However, FDPs from *R*. *microplus* and *H*. *asiaticum* formed the same clade. The FDP from *D*. *andersoni* was distant from all the FDPs analyzed in this study (Fig 2C). Furthermore, phylogenetic analysis showed that, cystatin of *O*. *turicata americanus*, *O*. *parkeri* and *O*. *coriaceus* fall within the same clade (Fig 2D). However, cystatin from *O*. *moubata* was close to the clade formed by *I*. *scapularis* and *R*. *microplus* (Fig 2D). Cystatins from *H*. *longicornis* and *D*. *andersoni* formed a different clade distinct from all the other analyzed cystatins (Fig 2D). The phylogenetic analysis of MLD revealed that, *O*. *turicata americanus*, *O*. *parkeri* and *O*. *coriaceus* MLD fall within the same clade and were close to the clade formed by *I*. *scapularis* and *I*. *persulcatus* (Fig 2E). However, MLD from *R*. *microplus*, *D*. *silvarum* and *D*. *andersoni* form divergent clades from the other analyzed MLDs (Fig 2E). Taken together, these analyses reveal that *O*. *turicata americanus* Toll, Lp, FDP, cystatin and MLD sequences were closer to their counterparts from other *Ornithodoros* species than to the protein sequences from hard ticks.

### Analysis of *O*. *turicata americanus toll*, *lp*, *cystatin*, *fdp* and *mld* expression in different tick developmental stages

**Fig 3:**
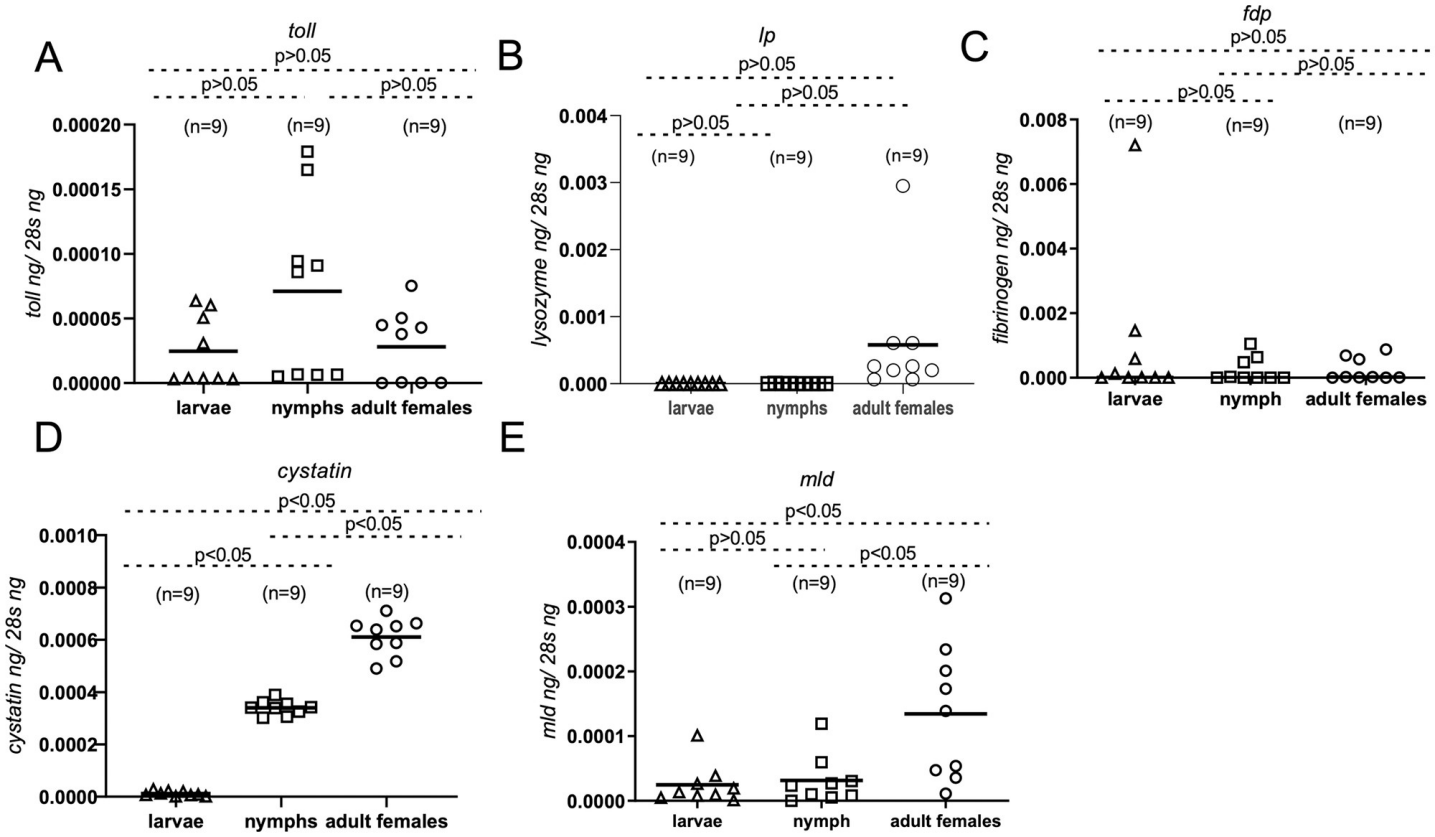
*Ornithodoros turicata americanus* innate immune genes show variable expression in different tick developmental stages. QRT-PCR analysis showing expression of *O*. *turicata americanus toll* (A) *lp* (B), *fdp* (C) *cystatin* (D) and *mld* (E) genes in different tick developmental stages of uninfected unfed ticks. The mRNA levels of innate immune genes were normalized to tick 28S rRNA levels. Open triangle represents larvae, open square represents nymphs and open circle represents adult *O*.*turicata americanus* female ticks. Each square or circle represents one tick and each triangle represents pool of 5 larvae. n indicates number of ticks per sample. P value from Student’s t-test is shown.

Expression of *O*. *turicata americanus* genes were analyzed in unfed larvae, nymphs, and adult female ticks (Fig 3). Quantitative RT-PCR (QRT-PCR) analysis revealed no significant (P>0.05) differences in the expression of *toll*, *lp* and *fdp* transcripts between larvae, nymphs, or adult females (Fig 3A–3C). The transcript levels of cystatin were noted to be significantly (P<0.05) higher in adults when compared to the levels noted in larvae or nymphs (Fig 3D). In addition, transcript levels of cystatin were noted to be significantly (P<0.05) higher in nymphs when compared to the levels noted in larvae (Fig 3D). The transcript levels of *mld* were noted to be significantly (P<0.05) higher in adults when compared to the levels noted in larvae or nymphs (Fig 3E). No significant (P>0.05) difference in the expression of *mld* was noted between larvae and nymphs (Fig 3E). These results show variable levels of *toll*, *lp*, *cystatin*, *and mld* transcript levels in different developmental stages of *O*. *turicata americanus* ticks.

### Blood feeding induces expression of *O*. *turicata americanus lp* gene in whole ticks

**Fig 4:**
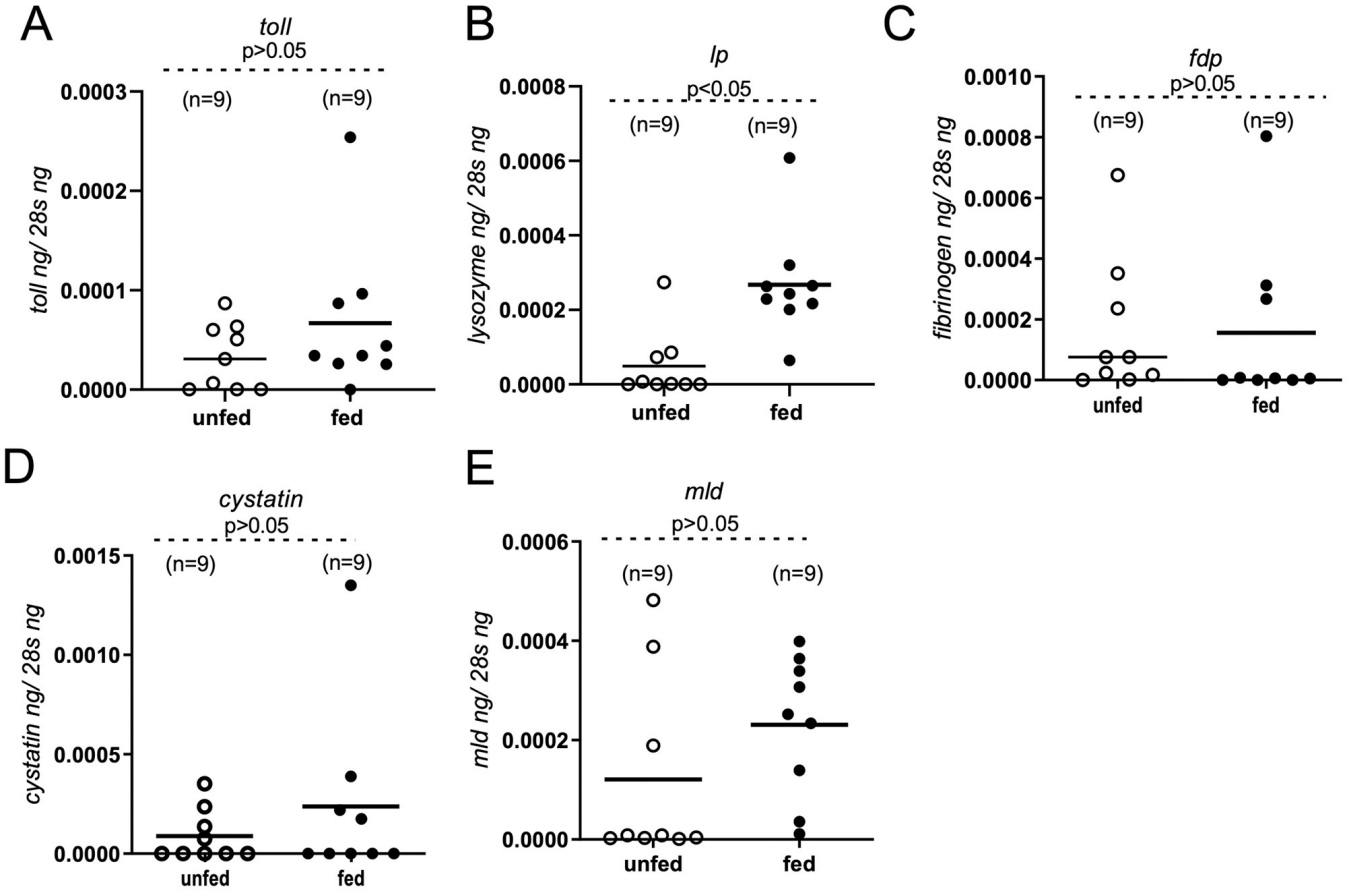
*Ornithodoros turicata americanus* innate immune gene expression upon tick blood feeding. QRT-PCR analysis showing expression of *O*. *turicata americanus toll* (A) *lp* (B), *fdp* (C) *cystatin* (D), and *mld* (E) genes in unfed and fed adult female ticks. Open circles represent unfed and closed circles represent fed adult female ticks. Each circle represents one tick. The mRNA levels of immune genes were normalized to tick 28S rRNA levels. n indicates number of ticks per sample. P value from Student’s t-test is shown.

To analyze whether blood feeding has any impact on *O*. *turicata americanus* immune gene expression, we assessed the levels of *toll*, *lp*, *fdp*, *cystatin*, *and mld* transcripts in unfed or fed ticks (Fig 4). We first analyzed whether blood feeding has any impact on the expression of house-keeping gene, 28S rRNA. QRT-PCR analysis revealed no change in the expression of 28S rRNA upon tick blood feeding (S8A Fig). Furthermore, QRT-PCR analysis revealed no significant differences in the expression of *O*. *turicata toll* (Fig 4A), *fdp* (Fig 4C) *cystatin* (Fig 4D) and *mld* (Fig 4E) between unfed and fed ticks. However, we noted significantly (P<0.05) higher levels of *lp* transcripts (Fig 4B) in fed ticks in comparison to the levels noted in unfed control ticks. These results show that blood feeding induces the expression of the *O*. *turicata americanus lp* gene.

### Blood feeding induces some of the *O*. *turicata americanus* immune genes in salivary glands and the midgut

**Fig 5:**
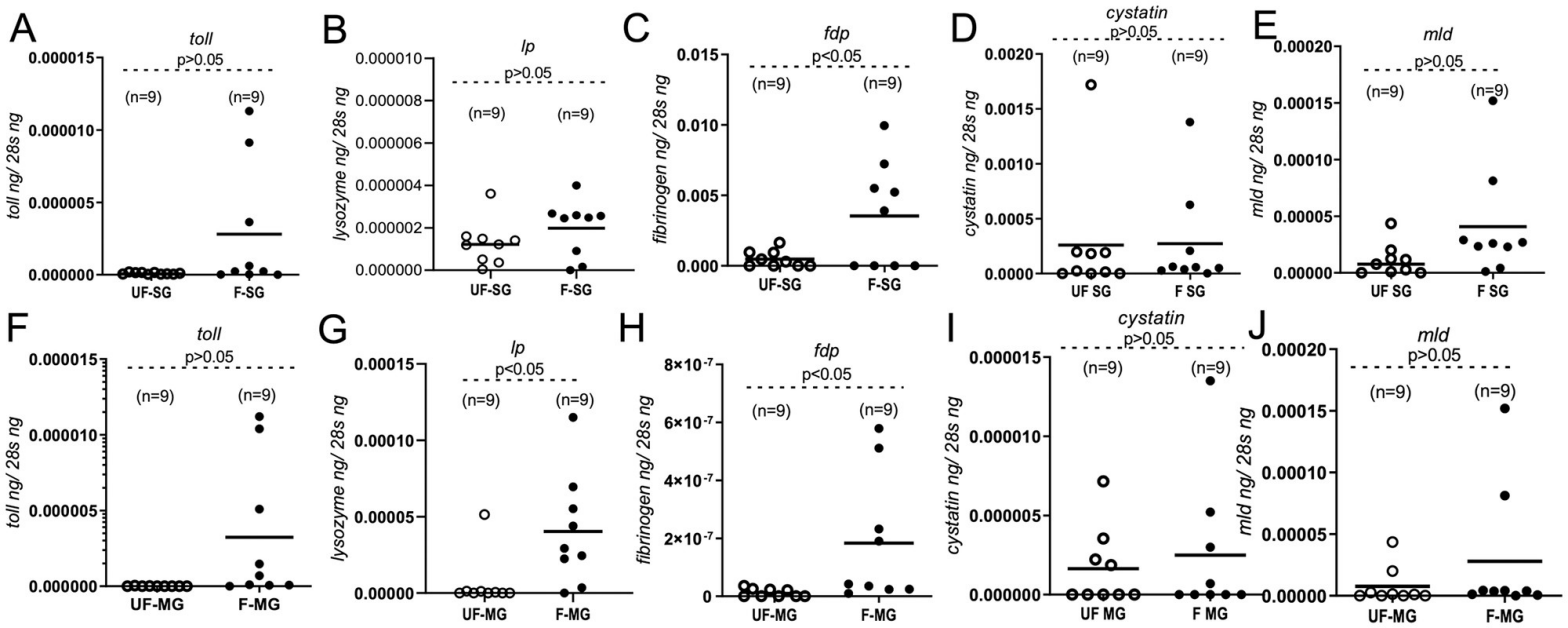
*Ornithodoros turicata americanus* innate immune gene expression in salivary glands and midgut upon tick feeding. QRT-PCR analysis showing expression of *O*. *turicata americanus toll* (A, F) *lp* (B, G), *fdp* (C, H) *cystatin* (D, I)), and *mld* (E, J) genes in salivary glands (SG) (A-E) or midgut (MG) (F-J) isolated from unfed (UF) or fed (F) adult female ticks. The mRNA levels of tick innate immune genes were normalized to tick 28S rRNA levels. Open circles represent data from unfed salivary glands or gut samples and closed circles represent data from fed salivary glands or gut samples. Each circle represents a pair of salivary glands or midgut isolated from one tick. n indicates number of ticks per sample. P-value from Student’s t-test is shown.

The observation of upregulation of *O*. *turicata americanus lp* gene expression in whole ticks prompted us to analyze all immune gene transcripts in the salivary gland (Fig 5A–5E) and midgut tissues (Fig 5F–5H). QRT-PCR analysis revealed no significant difference in the expression of *toll* (Fig 5A and 5F), *cystatin* (Fig 5D and 5I) *and mld* (Fig 5E and 5J) genes between unfed and fed salivary gland or guts tissues. However, significantly increased *fdp* transcript levels were evident in both salivary gland and midgut tissues isolated from fed *O*. *turicata americanus* ticks in comparison to the levels noted in these tissues from unfed ticks (Fig 5C and 5H). No significant difference (P>0.05) in the *lp* gene expression was noted between unfed and fed salivary gland tissues (Fig 5B). However, we noted significantly (P<0.05) increased expression of *lp* transcripts in midgut tissues isolated from fed *O*. *turicata americanus* ticks compared to the levels noted in these tissues from unfed ticks (Fig 5G). These results suggest a role for some of the *O*. *turicata americanus* immune genes in salivary glands and/or gut tissues during tick blood feeding.

### Subolesin plays a role in blood feeding of *O*. *turicata americanus* ticks

**Fig 6:**
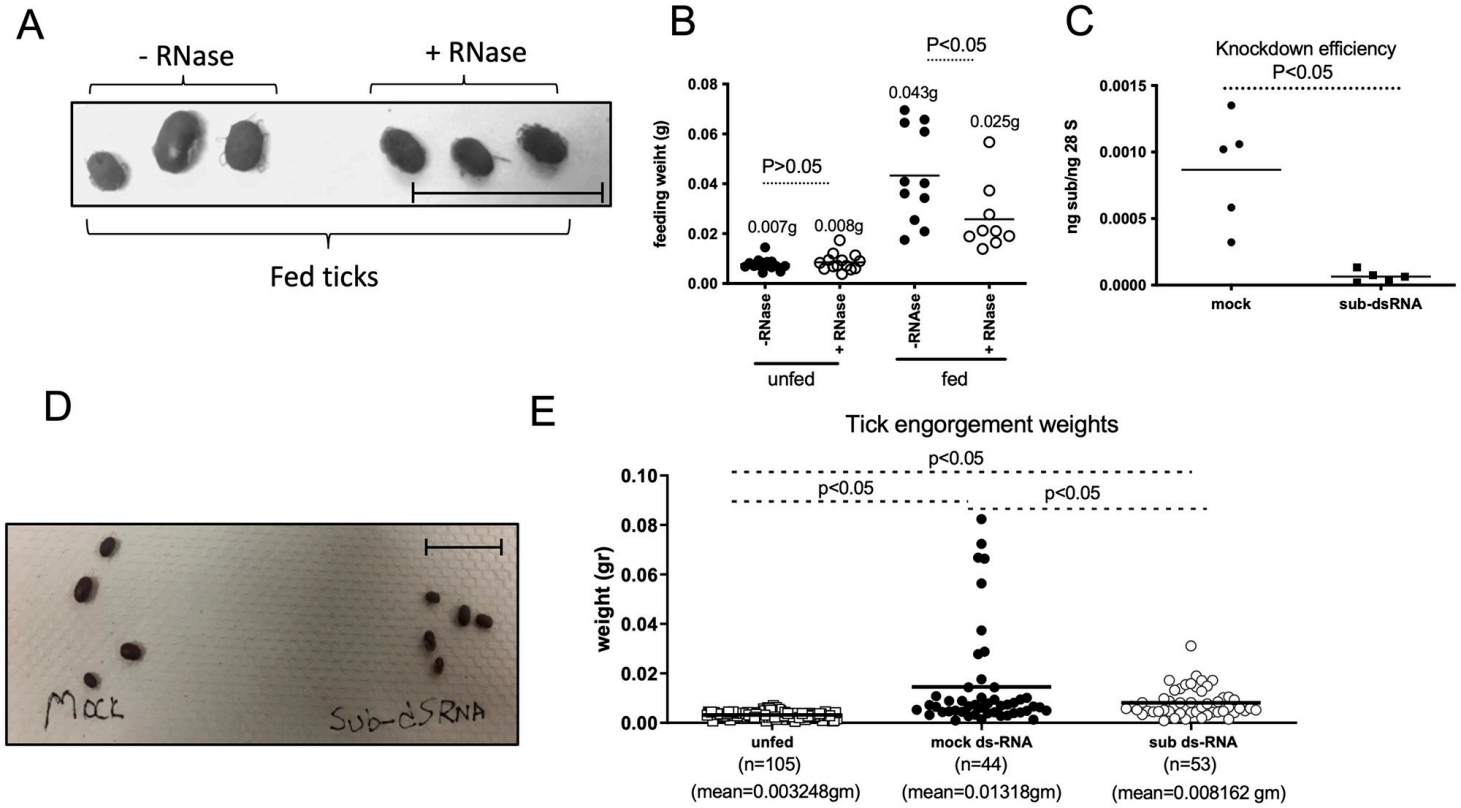
Knockdown of *O*. *turicata americanus* subolesin affects blood feeding by soft ticks. Photograph (A) and engorgement body weights (B) of *O*. *turicata americanus* female ticks fed *in vitro* on sheep blood with or without RNase is shown. Average weights are shown at the top of the scatter plot. QRT-PCR analysis of subolesin gene expression (C) photograph (D) and engorgement weights (E) of *O*. *turicata americanus* female ticks fed *in vitro* on blood containing mock-dsRNA or subolesin-dsRNA is shown. The mRNA levels of tick innate immune genes were normalized to tick 28S rRNA levels. Each circle or square represent data from one tick. Mean of the engorgement weights are shown either at the top of the circles (B) or at the bottom of the X-axis (E). P-value from Student’s t-test is shown. n indicates number of ticks per sample. ns indicates not significant.

In our previous study, we reported the use of *in vitro* blood feeding method to analyze gene expression in *O*. *turicata americanus* ticks [37]. To establish this method now for RNAi-mediated silencing of gene expression in these ticks, we first fed unfed adult female *O*. *turicata americanus* ticks *in vitro* on blood containing RNase. *Ornithodoros turicata americanus* ticks fed on blood containing RNase showed significantly (P<0.05) reduced engorgement weight when compared to the weight noted in ticks fed on blood without RNase (Fig 6A and 6B). These results show that the *in vitro* feeding method can be used to target RNA in these soft ticks. We then assessed whether targeting RNA by dsRNA also silence tick gene expression in these ticks. *Ornithodoros turicata americanus* ticks were fed on blood containing either mock- or *subolesin*-dsRNA (S8B Fig). QRT-PCR analysis revealed significant (P<0.05, approximately 90% knockdown efficiency) reduction in the *subolesin* transcripts in ticks fed on *sub*-dsRNA-containing blood when compared to the levels noted in ticks fed on mock-dsRNA-containing blood (Fig 6C). Furthermore, measurement of *O*. *turicata americanus* engorgement weights revealed a significant (P<0.05) reduction in the overall weight of ticks fed on *sub*-dsRNA-containing blood when compared to the weights noted in ticks fed on mock-dsRNA-containing blood (Fig 6D and 6E). As expected, significant (P<0.05) differences in the body weights were noted for ticks fed on sub-dsRNA or mock-dsRNA compared to weights noted for unfed controls (Fig 6D and 6E). Collectively, these results not only show feasibility on the use of *in vitro* feeding to silence tick gene expression in soft ticks but also suggests a role for *O*. *turicata americanus* subolesin in blood feeding.

### Subolesin regulates immune gene expression in *O*. *turicata americanus* ticks

**Fig 7:**
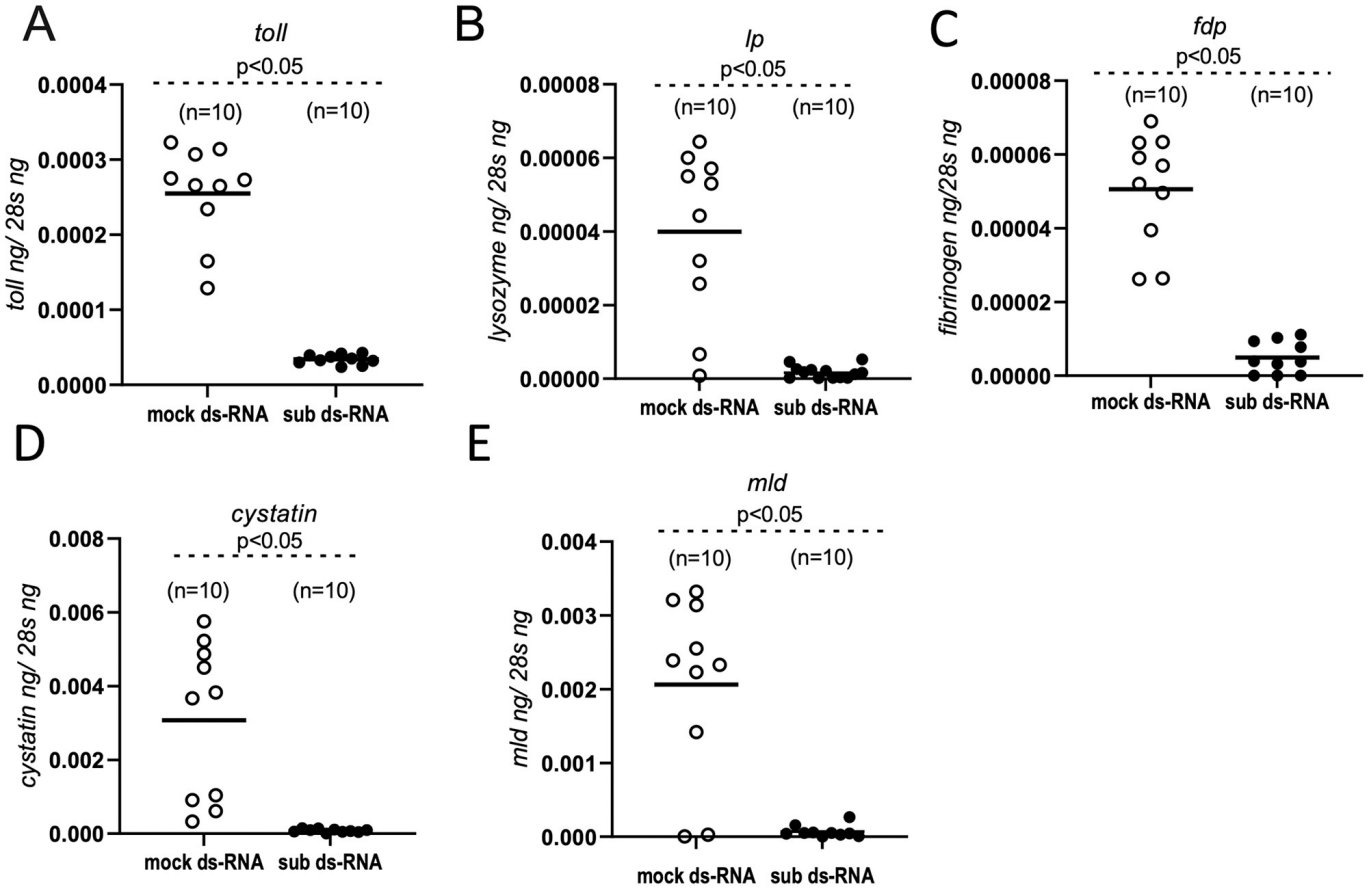
RNAi mediated silencing of subolesin causes significant downregulation of tick innate immune genes. QRT-PCR analysis showing expression of *O*. *turicata americanus toll* (A) *lp* (B), *fdp* (C) *cystatin* (D) and *mld* (E) genes in mock-dsRNA or subolesin-dsRNA-treated fed adult female ticks. The mRNA levels of tick innate immune genes are normalized to tick 28S rRNA levels. In all panels, closed circles represent mock-dsRNA-treated group and open circles represent *sub*-dsRNA treated ticks. Each circle represents one tick. n indicates number of ticks per sample. P-value from Student’s t-test is shown.

The role of subolesin in gene regulation is reported from hard ticks [20]. However, evidence on the role of subolesin in regulating gene expression in soft ticks is limited. We therefore determined whether knockdown of subolesin gene expression with dsRNA-treatment influences the expression of innate immune genes in soft ticks (Fig 7). QRTPCR analysis revealed that expression of *toll* (Fig 7A), *lp* (Fig 7B), *fdp* (Fig 7C), *cystatin* (Fig 7D) and *mld* (Fig 7E) genes were significantly downregulated in ticks fed on *sub*-dsRNA-containing blood in comparison to the levels noted in ticks fed on mock-dsRNA-containing blood. However, the transcript levels of *O*. *turicata americanus lipocalin* gene, *otlip*, were unaltered in ticks fed on mock- or *sub*-dsRNA ticks (S8C Fig). These results show that knockdown of subolesin results in the downregulation of some of the important tick innate immune genes. Taken together, our results show that *O*. *turicata americanus* subolesin is not only important in blood feeding but also in the regulation of some aspects of innate immune gene expression in these ticks.

## Discussion

Several studies have provided important information on the role of subolesin in blood feeding and gene expression in hard ticks [16–18, 20]. However, very little is known on the role of this molecule in soft ticks. This could be understood due to the lack of complete genome information in soft ticks. In this study, we combed previously reported transcriptomic data from *O*. *turicata americanus* [36] and transcript data from other *Ornithodoros* species and identified some of the transcripts that encode innate immune proteins in these soft ticks. In addition, our study noted that subolesin is not only important for *O*. *turicata americanus* blood feeding but also acts as an upstream activator of innate immune gene expression in these ticks.

The amino acid sequences of the orthologs of several innate immune genes of *O*. *turicata americanus* were found to be in the same clade shared by their counterparts from *Ornithodoros* species such as *O*. *moubata*, *O*. *parkeri and O*. *coriaceus* suggesting a conserved role of these genes in different soft ticks. The lower percentage identity of some of the *O*. *turicata* americanus innate immune genes with other *Ornithodoros* orthologs could be due to the analysis of partial sequence data. The levels of *O*. *turicata americanus cystatin* and *mld* transcripts were found to be significantly higher in adult female ticks compared to the levels noted in larval or nymphal ticks. The transcript levels of *mld* were variable within the same group of ticks. This could be due to biological transcriptional variations in different individual ticks. Unlike hard ticks, *Ornithodoros* adult female ticks can feed and mate multiple times and some pathogens can be transovarially transmitted in these soft ticks [6]. Therefore, it is not surprising to hypothesize that high expression of these innate immune genes in adult ticks would be necessary to keep a check on pathogen loads during each feeding and mating phases. Future studies may unravel the importance of these genes in *Ornithodoros* tick-pathogen interactions.

Tick fibrinogen-domain-containing proteins are lectin-type molecules involved in arthropod innate immunity [38]. Lectins have high affinity to bind to sialic acid, N-acetyl D-glucosamine and galactose [38,39]. Several lectin family proteins have been identified that are expressed in salivary glands, midgut and hemolymph [38]. Lectins such as Dorin M from *O*. *moubata* has been shown to have strong hemagglutinating activity [40]. The *O*. *turicata americanus* FDP shows approximately 52% identity with Dorin M. The increased transcription of *O*. *turicata americanus fdp* in salivary glands and midgut upon tick blood feeding suggests similar role for this protein in hemagglutination and/or in innate immunity of these ticks.

Based on the bioinformatic analysis, we previously noted that *O*. *turicata americanus* subolesin lacks a signal peptide and evidence of transmembrane domain suggesting its intracellular localization [28]. Therefore, subolesin is basically a cytosolic and a nuclear protein. We previously noted presence of a nuclear localization signal in *O*. *turicata americanus* subolesin [28] suggesting its role in the transcriptional regulation of gene expression. The finding of significantly reduced expression of *O*. *turicata americanus toll*, *lp*, *fdp*, *cystain* and *mld* in *sub*-dsRNA-treated ticks compared to the levels noted in mock-dsRNA-treated ticks clearly indicates its role in gene regulation in these soft ticks. Our current finding along with other published studies [17,18,20], strongly indicates a conserved role for subolesin in gene regulation in both hard and soft ticks.

RNAi interference is a widely used technique in addressing the role of tick genes in blood feeding and pathogen interactions [16,17,20,35,41–50]. Our study provided evidence for the use of an *in vitro* feeding system to knockdown gene expression in *O*. *turicata americanus* ticks. We noted eight-fold less levels of subolesin transcripts in the sub-dsRNA-treated ticks compared to the levels noted in mock-dsRNA treated ticks. We believe that achieving good silencing efficiency of the tick genes via RNAi interference depends on several factors including but not limited to quality of the double stranded RNA, concentration of dsRNA, route of treatment and developmental stage of ticks. In our experimental set up, we used 1μg/ml mock- or *sub*-dsRNA. We have not tested whether lower than 1μg/ml dose of dsRNA could also result in good silencing efficiency of gene expression in these ticks. Future studies could address whether lower amounts of dsRNA can also be used to silence gene expression in these ticks.

Subolesin has been shown to play an important role in tick reproduction, blood feeding and interactions with pathogens [16–18,20,51]. The observation of reduced engorgement weight of *O*. *turicata americanus* sub-dsRNA-treated ticks when compared to the weight noted from mock-dsRNA-treated ticks indicates an important role of subolesin in blood feeding in these ticks. This observation supports previous studies that addressed the role of subolesin in blood ingestion and feeding in hard ticks [16,17,20]. Several studies have indicated subolesin as a universal candidate for the development of anti-tick vaccine to target multiple ticks [18,51,52]. Furthermore, studies have reported that antibodies can enter cultured tick cells [51,53,54]. Therefore, the hypothesis of antibodies targeting subolesin entering tick cells and blocking its intracellular function cannot be ruled out. *Ornithodoros* ticks are important vectors for various human pathogens including relapsing fever agents. Due to the observed role of subolesin in *O*. *turicata americanus* blood feeding and control of innate immune gene expression, our current study strongly supports previous findings [18,51,52] and indicates that development of anti-subolesin based strategies could also be effective to target soft ticks.

In summary, our study not only indicates that subolesin is important in *O*. *turicata americanus* blood feeding but also plays an important role in the activation of innate immune gene expression in these ticks. Studies like these are important to understand the conserved role of potential anti-tick vaccine candidate molecules, such as subolesin, in blood feeding and gene expression.

## Methods

### Ethics statement

All animal work in the study was performed based on the Old Dominion University Institutional animal care and use committee (IACUC) approved animal protocol (10–018). During tick feeding, Acepromazine was used as a tranquilizer to minimize discomfort/anxiety in animals.

### Soft ticks and mice

In this study laboratory reared uninfected *Ornithodoros turicata americanus* ticks were used. Different developmental stages of *O*. *turicata americanus* including larvae, nymphs and adult female ticks were used in this study. The original *O*. *turicata americanus* ticks were collected from the burrows of the gopher tortoise (*Gopherus polyphemus*) in Florida, U.S.A. [donated by Dr. James H. Oliver, Jr., Georgia Southern University, Statesboro, GA to Dr. Daniel Sonenshine, Old Dominion University (ODU), VA] and maintained as described [55]. Unfed ticks were fed on naïve 6–8 weeks old CD1 mice (Charles River laboratories) to generate fed ticks. Ticks were fed to repletion after which they were collected and processed for RNA extractions. *Ornithodoros turicata americanus* ticks were housed in a controlled environment chamber (Parameter Generation and Control, Black Mountain, NC) set at 23°C with 14/10 h light/dark cycles and 93% relative humidity.

### Polymerase chain reaction (PCR), cloning and sequencing of *O*. *turicata americanus* genes

Total RNA extractions followed by cDNA synthesis was generated from unfed or fed *O*. *turicata americanus* ticks as described [28,56]. The synthesized cDNA was used as a template for amplification of fibrinogen-domain containing salivary protein (*fdp*), *cystatin* and ML-domain-containing protein (*mld*) gene fragments. Oligonucleotides to amplify these genes are shown in S1 Table. PCR was performed using the following conditions-initial denaturation at 94 degrees for 3 mins followed by 32 cycles of steps including 94 degrees for 1 min,54 degrees for 1 min and 72 degrees for 2 mins. PCR reactions were later run on 1.2% agarose gels and corresponding bands for *fdp* (~567 bp), *cystatin* (~351 bp) and *mld* (~687 bp) gene fragments were purified using Qiagen Gel Extraction Kit (Qiagen,USA). The PCR product was later ligated into pGEMT vector (Promega, USA) and transformed into *E*. *coli* JM109 competent cells. The transformed JM109 cells were then plated on Luria-Bertani (LB) agar plates containing ampicillin (50 micrograms/ml) and the respective clones for *fdp*, *cystatin* and *mld* genes were selected for plasmid preparation. Plasmids were purified using Qiagen mini prep kit (Qiagen). At least three independent positive clones were sequenced from both ends at the Simple Seq core facility (Eurofins MWG Operon Inc., Huntsville, USA) using oligonucleotides shown in S1 Table. The alignment of nucleotide and amino acid sequences of all three clones for each gene are shown as S2–S5 Figs.

### Sequence alignment and phylogenetic analysis

The sequence alignment and phylogenetic analysis was performed as described [43]. The following are the GenBank Accession numbers for Toll sequences: KAG0432202.1-*Ixodes persulcatus*, KAH6927628.1- *Hyalomma asiaticum*, XP_029821922.2-*Ixodes scapularis*, XP_037282253.1- *Rhipicephalus microplus*, XP_037520387.1- *Rhipicephalus sanguineus*, XP_037577366.1- *Dermacentor silvarum*, XP_050026248.1- *Dermacentor andersoni*. The GenBank Accession numbers for Lysozyme precursor are: AAL17868.1-*O*. *moubata*, KAH8019263.1-*R*. *microplus*, XP_029836071.4-*I*. *scapularis*, XP_037283720.1-*R*. *microplus*, XP_037554643.1-*D*. *silvarum*, XP_050046369.1-*D*. *andersoni*. The GenBank accession numbers for FDP are: AAP93589.1-*O*. *moubata*, ABR23368.1-*Ornithodoros parkeri*, KAH6934233.1-*H*. *asiaticum*, KAH9377322.1-*H*. *longicornis*, XP_037278167.1-*R*. *microplus*, XP_040076131.1-*I*. *scapularis*, XP_050029118.1-*D*. *andersoni*. The GenBank accession numbers for cystatin are: AAS55948.1-*O*. *moubata*, ABC94582.1-*Haemaphysalis longicornis*, ABR23498.1-*O*. *parkeri*, ACB70345.1-*O*. *coriaceus*, AGW80658.1-*R*. *microplus*, EEC07265.1-*I*. *scapularis*, XP_050037659.1-*D*. *andersoni*. The GenBank accession numbers for MLD are: ABR23350.1-*O*. *parkeri*, ACB70390.1-*O*. *coriaceus*, EEC18444.1-*I*. *scapularis*, KAG0424161.1-*I*. *persulcatus*, XP_037274314.1-*R*. *microplus*, XP_037575527.1-*D*. *silvarum*, XP_050036812.1-*D*. *andersoni*. The GenBank accession numbers for *O*. *turicata americanus* are: Toll-GDIC01007985.1, Lysozyme precursor- GDIC01003173.1, *cytatin* (OQ330852), *fdp* (OQ330853) and *mld* (OQ330854). Nucleotide or amino acid sequences were downloaded from GenBank and analyzed with MegAlign Pro (v17.3.1) in DNASTAR software. Phylogenetic trees were constructed using BIONJ (Neighbor-joining) method in DNASTAR.

### RNA extractions, cDNA synthesis and Quantitative Real-time PCR (QRT-PCR) Analysis

Total RNA from larvae, nymphal, adult females, mock- or *sub*-dsRNA-treated ticks was generated using the BioRad Aurum Total RNA Mini kit following the manufacturer’s instruction. cDNA synthesis was carried out using iSCRIPT cDNA synthesis kit (BioRAD, USA). The cDNA was then used as a template to quantify transcript levels of five *O*. *turicata americanus* immune genes that includes *toll*, lysozyme precursor (*lp*), fibrinogen domain-containing protein (*fdp*), *cystatin*, and ML-domain protein (*mld*). As an internal control and to normalize the amount of template, *O*. *turicata americanus* 28S rRNA levels were quantified. QRT-PCR was performed using CFX96 QPCR machine (BioRad, USA) and iQ-SYBR Green Supermix (BioRad, USA) as described [44,56]. In QRT-PCR reactions, the standard curve was generated using 10-fold serial dilutions starting from 1 ng to 0.00001 ng of known quantities of respective fragments.

### Isolation of salivary glands and midgut from *O*. *turicata americanus*

Pairs of salivary glands and midgut from unfed or fed nymphal soft ticks were dissected in sterile Ix PBS (phosphate buffered saline) as described [55]. The tissues were processed for homogenization in lysis buffer (BioRad, Aurum total RNA kit) and total RNA extractions were performed using Aurum total RNA kit (BioRad, USA) in accordance with the manufacturer’s instructions. The extracted RNA was reverse transcribed to cDNA using iSCRIPT cDNA kit (BioRad, USA) and used for QRT-PCR analysis.

### ds-RNA synthesis and RNAi-mediated knockdown of gene expression in *O*. *turicata americanus* ticks

The pGEMT-subolesin-plasmid [28] was used as a template to amplify DNA encoding a fragment of subolesin for dsRNA synthesis. The subolesin gene-specific primers containing T7 promoters were used in PCR. The oligonucleotides used in the amplification are listed in S1 Table. Mock-dsRNA was prepared from the multiple cloning site (MCS) region of empty pL4440 vector as described [44,56]. The dsRNAs complementary to pL4440 MCS region or subolesin gene sequences were synthesized using the MEGAscript RNAi Kit (Ambion Inc., USA) and following manufacturer’s instructions. For RNA interference, we followed *in vitro* feeding techniques as published in our previous work [37]. Briefly, adult female ticks were fed *in vitro* on 1.5 ml of defibrinated sheep blood (ThermoFisherScientific, USA) containing 2mg/ml sucrose, 1mM ATP and 1μg/ml mock- or *sub*-dsRNA with or without 1μg/ml RNase in 15 ml tubes as described [37]. The body weights of individual unfed nymphal or adult female ticks were determined before feeding was initiated and after feeding using an analytical balance (Sartorius, USA). Two to three *O*. *turicata americanus* adult female ticks were placed in 5ml sterilized plastic tubes with the open end sealed with a layer of parafilm. The sealed tubes were then inverted and inserted into the 15-ml tubes. Fresh blood was used in all blood feeding experiments. The *in vitro* feeding apparatus was placed in a water bath set at 37°C for 45 mins. Ticks fed through the parafilm membrane and engorged on the blood. After feeding, ticks were collected, washed with sterile distilled water, surface-sterilized with 70% ethanol, air-dried and were housed in the environmental chamber for 16–20 h. RNA from mock- or *sub*-dsRNA-treated ticks was extracted from fed ticks. Quantitative real-time PCR analysis was performed with cDNA to determine knockdown efficiency.

### Statistical analysis

Statistical significance was analyzed using GraphPad Prism software and Microsoft Excel 2010. To compare two means, the non-paired Student t-test was performed. P values of < 0.05 were considered significant for analysis. Horizontal bars in the graphs represent average of the mean value. Wherever it was required, statistical test and P values used are reported.

## Supporting information

S1 Fig PCR amplification of larger size PCR fragments of *O*. *turicata americanus fdp*, *cystatin* and *mld*. Agarose gel image showing the PCR products of *O*. *turicata americanus fdp* (A), *cystatin* (B), and *mld* (C) gene fragments from cDNA samples generated from unfed adult female ticks. M indicates marker and NTC indicates no template control.
(TIF)

S2 Fig Nucleotide sequence alignment of *O*. *turicata americanus fdp* sequences from three clones. The nucleotide sequence alignment performed using DNA MegAlign software of three clones of *O*. *turicata americanus fdp* is shown. Matched sequences are shaded with black color. Consensus sequences are shown below the ruler. Ruler represents nucleobase number.
(TIF)

S3 Fig Nucleotide sequence alignment of *O*. *turicata americanus cystatin* sequences from three clones. The nucleotide sequence alignment performed using DNA MegAlign software of three clones of *O*. *turicata americanus cystatin* is shown. Matched sequences are shaded with black color. Consensus sequences are shown below the ruler. Ruler represents nucleobase number.
(TIF)

S4 Fig Nucleotide sequence alignment of *O*. *turicata americanus mld* sequences from three clones. The nucleotide sequence alignment performed using DNA MegAlign software of three clones of *O*. *turicata americanus mld* is shown. Matched sequences are shaded with black color. Consensus sequences are shown below the ruler. Ruler represents nucleobase number.
(TIF)

S5 Fig Amino acid sequence alignment of *O*. *turicata americanus* FDP, cystatin and MLD sequences from three clones. The annotated amino acid sequence alignment performed using DNA MegAlign software of three clones of *O*. *turicata americanus* FDP, cystatin and MLD is shown. Matched sequences are shaded with black color. Consensus sequences are shown below the ruler. Ruler represents amino acid number.
(TIF)

S6 Fig Amino acid sequence identity between *O*. *turicata americanus* TOLL, Lp and FDP with orthologs from other ticks. The percent identity (horizontally above black boxed diagonal line) and distance (vertically below black boxed diagonal line) of the *O*. *turicata americanus* TOLL, Lp and FDP amino acid sequence in comparison to the ortholog proteins from other hard and soft ticks is shown. The data of percent identity and distance was generated using DNASTAR MegAlign software. GenBank accession numbers and species names are indicated.
(TIF)

S7 Fig Amino acid sequence identity between *O*. *turicata americanus* cystatin and MLD with orthologs from other ticks. The percent identity (horizontally above black boxed diagonal line) and distance (vertically below black boxed diagonal line) of the *O*. *turicata americanus* cystatin and MLD amino acid sequence in comparison to the ortholog proteins from other hard and soft ticks is shown. The data of percent identity and distance was generated using DNASTAR MegAlign software. GenBank accession numbers and species names are indicated.
(TIF)

S8 Fig Synthesis of *subolesin*-dsRNA. A) QRT-PCR analysis showing levels of *O*. *turicata americanus* 28S rRNA transcripts normalized to total RNA in samples generated from unfed and fed adult female ticks (A), unfed larvae, nymphs, and adults (B), salivary glands (C) and midgut (D) isolated from unfed and fed adult female ticks. E) Agarose gel image showing generation and purification of subolesin dsRNA using MegScript RNAi kit is shown. M indicates marker and elution numbers (1,2) are indicated. Arrow indicates sub-dsRNA fragment. F) QRT-PCR analysis showing levels of *O*. *turicata americanus lipocalin* gene transcripts normalized to 28S rRNA. In A and C, each circle represents one tick. n indicates number of ticks per sample. P-value from Student’s t-test is shown.
(TIF)

S1 Table Oligonucleotides used in this study. Oligonucleotides used in this study are listed in this table.
(TIF)

## References

[1] Lazzari CR, Fauquet A, Lahondère C, Araújo RN, Pereira MH. Soft ticks perform evaporative cooling during blood-feeding. Journal of insect physiology. 2021;130:104197. doi: 10.1016/j.jinsphys.2021.104197 33545105

[2] Estrada-Peña A, Jongejan F. Ticks feeding on humans: a review of records on human-biting Ixodoidea with special reference to pathogen transmission. Experimental & applied acarology. 1999;23(9):685–715. doi: 10.1023/a:1006241108739 10581710

[3] Boulanger N, Boyer P, Talagrand-Reboul E, Hansmann Y. Ticks and tick-borne diseases. Medecine et maladies infectieuses. 2019;49(2):87–97. doi: 10.1016/j.medmal.2019.01.007 30736991

[4] Sarwar M. Status of argasid (soft) ticks (Acari: Parasitiformes: Argasidae) in relation to transmission of human pathogens. International Journal of Vaccines and Vaccination. 2017;4(4):00089.

[5] Hoskins JD. Ixodid and argasid ticks: keys to their identification. Veterinary Clinics of North america: small animal Practice. 1991;21(1):185–97. 2014622 10.1016/s0195-5616(91)50018-8

[6] Sonenshine DE, Roe R. Biology of Ticks, Second Edition. Oxford University Press. 2014;2.

[7] Anderson JF, Magnarelli LA. Biology of ticks. Infectious disease clinics of North America. 2008;22(2):195–215. doi: 10.1016/j.idc.2007.12.006 18452797

[8] Apanaskevich DA, Oliver J, Sonenshine D, Roe R. Life cycles and natural history of ticks. Biology of ticks. 2013;1:59–73.

[9] Mccoy KD, Léger E, Dietrich M. Host specialization in ticks and transmission of tick-borne diseases: a review. Frontiers in cellular and infection microbiology. 2013;3:57. doi: 10.3389/fcimb.2013.00057 24109592 PMC3790072

[10] George JE, Pound, J.M., Davey, R.B. Acaricides for controlling ticks on cattle and the problem of acaricide resistance. In: Bowman, AS, Nuttall, P (Eds), Ticks: Biology, Disease and Control Cambridge University Press, Cambridge. 2008:408–23.

[11] Contreras M, Kasaija PD, Kabi F, Mugerwa S, De la Fuente J. The Correlation between Subolesin-Reactive Epitopes and Vaccine Efficacy. Vaccines (Basel). 2022;10(8). Epub 2022/08/27. doi: 10.3390/vaccines10081327 36016215 PMC9414912

[12] de la Fuente J, Contreras M. Additional considerations for anti-tick vaccine research. Expert Rev Vaccines. 2022;21(8):1019–21. Epub 2022/04/28. doi: 10.1080/14760584.2022.2071704 . 35475778

[13] Neelakanta G, Sultana H. Transmission-Blocking Vaccines: Focus on Anti-Vector Vaccines against Tick-Borne Diseases. Arch Immunol Ther Exp (Warsz). 2015;63(3):169–79. Epub 2014/12/17. doi: 10.1007/s00005-014-0324-8 25503555 PMC4429137

[14] Neelakanta G, Sultana H. Tick saliva and salivary glands: what do we know so far on their role in arthropod blood feeding and pathogen transmission. Frontiers in Cellular and Infection Microbiology. 2022:1430. doi: 10.3389/fcimb.2021.816547 35127563 PMC8809362

[15] Parthasarathi BC, Kumar B, Ghosh S. Current status and future prospects of multi-antigen tick vaccine. J Vector Borne Dis. 2021;58(3):183–92. Epub 2022/02/17. doi: 10.4103/0972-9062.321739 . 35170454

[16] de la Fuente J, Almazan C, Blas-Machado U, Naranjo V, Mangold AJ, Blouin EF, et al. The tick protective antigen, 4D8, is a conserved protein involved in modulation of tick blood ingestion and reproduction. Vaccine. 2006;24(19):4082–95. Epub 2006/04/04. doi: 10.1016/j.vaccine.2006.02.046 . 16580098

[17] de la Fuente J, Almazan C, Blouin EF, Naranjo V, Kocan KM. RNA interference screening in ticks for identification of protective antigens. Parasitology research. 2005;96(3):137–41. Epub 2005/04/13. doi: 10.1007/s00436-005-1351-5 . 15824899

[18] de la Fuente J, Moreno-Cid JA, Galindo RC, Almazan C, Kocan KM, Merino O, et al. Subolesin/Akirin vaccines for the control of arthropod vectors and vectorborne pathogens. Transboundary and emerging diseases. 2013;60 Suppl 2:172–8. Epub 2014/03/05. doi: 10.1111/tbed.12146 . 24589118

[19] Artigas-Jeronimo S, Villar M, Cabezas-Cruz A, Valdes JJ, Estrada-Pena A, Alberdi P, et al. Functional Evolution of Subolesin/Akirin. Front Physiol. 2018;9:1612. Epub 2018/12/14. doi: 10.3389/fphys.2018.01612 30542290 PMC6277881

[20] De la Fuente J, Maritz-Olivier C, Naranjo V, Ayoubi P, Nijhof AM, Almazán C, et al. Evidence of the role of tick subolesin in gene expression. BMC genomics. 2008;9(1):1–16. doi: 10.1186/1471-2164-9-372 18673577 PMC2518936

[21] Carreon D, de la Lastra JM, Almazan C, Canales M, Ruiz-Fons F, Boadella M, et al. Vaccination with BM86, subolesin and akirin protective antigens for the control of tick infestations in white tailed deer and red deer. Vaccine. 2012;30(2):273–9. Epub 2011/11/15. doi: 10.1016/j.vaccine.2011.10.099 . 22079077

[22] Kasaija PD, Contreras M, Kabi F, Mugerwa S, de la Fuente J. Vaccination with Recombinant Subolesin Antigens Provides Cross-Tick Species Protection in Bos indicus and Crossbred Cattle in Uganda. Vaccines (Basel). 2020;8(2). Epub 2020/06/24. doi: 10.3390/vaccines8020319 32570925 PMC7350222

[23] Davis GE. Ornithodoros turicata:the males; feeding and copulation habits, fertility, span of life, and the transmission of relapsing fever spirochetes. Pub Health Rep. 1941;56:1799–802. doi: 10.2307/4583854

[24] Gaudreault NN, Madden DW, Wilson WC, Trujillo JD, Richt JA. African Swine Fever Virus: An Emerging DNA Arbovirus. Front Vet Sci. 2020;7:215. Epub 2020/06/02. doi: 10.3389/fvets.2020.00215 32478103 PMC7237725

[25] Burgdorfer W. The possible role of ticks as vectors of leptospirae. I. Transmission of Leptospira pomona by the argasid tick, Ornithodoros turicata, and the persistance of this organism in its tissues. Experimental parasitology. 1956;5(6):571–9. Epub 1956/11/01. doi: 10.1016/0014-4894(56)90030-3 . 13375683

[26] Boyle WK, Wilder HK, Lawrence AM, Lopez JE. Transmission dynamics of Borrelia turicatae from the arthropod vector. PLoS Negl Trop Dis. 2014;8(4):e2767. Epub 2014/04/05. doi: 10.1371/journal.pntd.0002767 24699275 PMC3974661

[27] Manzano-Román R, Díaz-Martín V, Oleaga A, Siles-Lucas M, Pérez-Sánchez R. Subolesin/akirin orthologs from Ornithodoros spp. soft ticks: cloning, RNAi gene silencing and protective effect of the recombinant proteins. Veterinary parasitology. 2012;185(2–4):248–59. doi: 10.1016/j.vetpar.2011.10.032 22105082

[28] Sultana H, Patel U, Sonenshine DE, Neelakanta G. Identification and comparative analysis of subolesin/akirin ortholog from Ornithodoros turicata ticks. Parasit Vectors. 2015;8:132. Epub 2015/04/19. doi: 10.1186/s13071-015-0749-x 25889484 PMC4359563

[29] Ibelli AM, Hermance MM, Kim TK, Gonzalez CL, Mulenga A. Bioinformatics and expression analyses of the Ixodes scapularis tick cystatin family. Exp Appl Acarol. 2013;60(1):41–53. Epub 2012/10/12. doi: 10.1007/s10493-012-9613-2 23053911 PMC4058331

[30] Simo L, Kazimirova M, Richardson J, Bonnet SI. The Essential Role of Tick Salivary Glands and Saliva in Tick Feeding and Pathogen Transmission. Front Cell Infect Microbiol. 2017;7:281. Epub 2017/07/12. doi: 10.3389/fcimb.2017.00281 28690983 PMC5479950

[31] Sterba J, Dupejova J, Fiser M, Vancova M, Grubhoffer L. Fibrinogen-related proteins in ixodid ticks. Parasit Vectors. 2011;4:127. Epub 2011/07/07. doi: 10.1186/1756-3305-4-127 21729260 PMC3141747

[32] Rego RO, Hajdusek O, Kovar V, Kopacek P, Grubhoffer L, Hypsa V. Molecular cloning and comparative analysis of fibrinogen-related proteins from the soft tick Ornithodoros moubata and the hard tick Ixodes ricinus. Insect Biochem Mol Biol. 2005;35(9):991–1004. Epub 2005/06/28. doi: 10.1016/j.ibmb.2005.04.001 . 15979000

[33] Horáčková J, Rudenko N, Golovchenko M, Havlíková S, Grubhoffer L. IrML–a gene encoding a new member of the ML protein family from the hard tick, Ixodes ricinus. Journal of Vector Ecology. 2010;35(2):410–8. doi: 10.1111/j.1948-7134.2010.00100.x 21175949

[34] Fogaca AC, Sousa G, Pavanelo DB, Esteves E, Martins LA, Urbanova V, et al. Tick Immune System: What Is Known, the Interconnections, the Gaps, and the Challenges. Front Immunol. 2021;12:628054. Epub 2021/03/20. doi: 10.3389/fimmu.2021.628054 33737931 PMC7962413

[35] Khanal S, Taank V, Anderson JF, Sultana H, Neelakanta G. Rickettsial Pathogen Perturbs Tick Circadian Gene to Infect the Vertebrate Host. Int J Mol Sci. 2022;23(7). Epub 2022/04/13. doi: 10.3390/ijms23073545 35408905 PMC8998576

[36] Egekwu N, Sonenshine DE, Garman H, Barshis DJ, Cox N, Bissinger BW, et al. Comparison of synganglion neuropeptides, neuropeptide receptors and neurotransmitter receptors and their gene expression in response to feeding in Ixodes scapularis (Ixodidae) vs. Ornithodoros turicata (Argasidae). Insect Mol Biol. 2016;25(1):72–92. Epub 2016/01/20. doi: 10.1111/imb.12202 . 26783017

[37] Neelakanta G, Sultana H, Sonenshine DE, Marconi RT. An In Vitro Blood-Feeding Method Revealed Differential Borrelia turicatae (Spirochaetales: Spirochaetaceae) Gene Expression After Spirochete Acquisition and Colonization in the Soft Tick Ornithodoros turicata (Acari: Argasidae). J Med Entomol. 2017;54(2):441–9. Epub 2017/04/12. doi: 10.1093/jme/tjw171 . 28399292

[38] Grubhoffer L, Kovar V, Rudenko N. Tick lectins: structural and functional properties. Parasitology. 2004;129 Suppl:S113–25. Epub 2005/06/09. doi: 10.1017/s0031182004004858 . 15938508

[39] Huang X, Tsuji N, Miyoshi T, Nakamura-Tsuruta S, Hirabayashi J, Fujisaki K. Molecular characterization and oligosaccharide-binding properties of a galectin from the argasid tick Ornithodoros moubata. Glycobiology. 2007;17(3):313–23. Epub 2006/11/25. doi: 10.1093/glycob/cwl070 . 17124195

[40] Kovar V, Kopacek P, Grubhoffer L. Isolation and characterization of Dorin M, a lectin from plasma of the soft tick Ornithodoros moubata. Insect Biochem Mol Biol. 2000;30(3):195–205. Epub 2000/03/25. doi: 10.1016/s0965-1748(99)00107-1 . 10732987

[41] Dahmani M, Anderson JF, Sultana H, Neelakanta G. Rickettsial pathogen uses arthropod tryptophan pathway metabolites to evade reactive oxygen species in tick cells. Cell Microbiol. 2020;22(10):e13237. Epub 2020/06/21. doi: 10.1111/cmi.13237 32562372 PMC7483324

[42] Mahesh PP, Namjoshi P, Sultana H, Neelakanta G. Immunization against arthropod protein impairs transmission of rickettsial pathogen from ticks to the vertebrate host. NPJ Vaccines. 2023;8(1):79. Epub 2023/05/31. doi: 10.1038/s41541-023-00678-y 37253745 PMC10229574

[43] Namjoshi P, Dahmani M, Sultana H, Neelakanta G. Rickettsial pathogen inhibits tick cell death through tryptophan metabolite mediated activation of p38 MAP kinase. iScience. 2023;26(1):105730. Epub 2022/12/31. doi: 10.1016/j.isci.2022.105730 36582833 PMC9792911

[44] Ramasamy E, Taank V, Anderson JF, Sultana H, Neelakanta G. Repression of tick microRNA-133 induces organic anion transporting polypeptide expression critical for Anaplasma phagocytophilum survival in the vector and transmission to the vertebrate host. PLoS Genet. 2020;16(7):e1008856. Epub 2020/07/03. doi: 10.1371/journal.pgen.1008856 32614824 PMC7331985

[45] Regmi P, Khanal S, Neelakanta G, Sultana H. Tick-Borne Flavivirus Inhibits Sphingomyelinase (IsSMase), a Venomous Spider Ortholog to Increase Sphingomyelin Lipid Levels for Its Survival in Ixodes scapularis Ticks. Front Cell Infect Microbiol. 2020;10:244. Epub 2020/07/14. doi: 10.3389/fcimb.2020.00244 32656091 PMC7325911

[46] Turck JW, Taank V, Neelakanta G, Sultana H. Ixodes scapularis Src tyrosine kinase facilitates Anaplasma phagocytophilum survival in its arthropod vector. Ticks Tick Borne Dis. 2019;10(4):838–47. Epub 2019/04/20. doi: 10.1016/j.ttbdis.2019.04.002 31000483 PMC6613393

[47] de la Fuente J, Kocan KM. The Impact of RNA Interference in Tick Research. Pathogens. 2022;11(8). Epub 2022/07/28. doi: 10.3390/pathogens11080827 35894050 PMC9394339

[48] Kim TK, Tirloni L, Berger M, Diedrich JK, Yates JR 3rd, Termignoni C, et al. Amblyomma americanum serpin 41 (AAS41) inhibits inflammation by targeting chymase and chymotrypsin. Int J Biol Macromol. 2020;156:1007–21. Epub 2020/04/23. doi: 10.1016/j.ijbiomac.2020.04.088 . 32320803 PMC11005088

[49] O’Neal AJ, Singh N, Rolandelli A, Laukaitis HJ, Wang X, Shaw DK, et al. Croquemort elicits activation of the immune deficiency pathway in ticks. Proc Natl Acad Sci U S A. 2023;120(20):e2208673120. Epub 2023/05/08. doi: 10.1073/pnas.2208673120 37155900 PMC10193931

[50] Shaw DK, Wang X, Brown LJ, Chavez AS, Reif KE, Smith AA, et al. Infection-derived lipids elicit an immune deficiency circuit in arthropods. Nat Commun. 2017;8:14401. Epub 2017/02/15. doi: 10.1038/ncomms14401 28195158 PMC5316886

[51] de la Fuente J, Moreno-Cid JA, Canales M, Villar M, de la Lastra JM, Kocan KM, et al. Targeting arthropod subolesin/akirin for the development of a universal vaccine for control of vector infestations and pathogen transmission. Veterinary parasitology. 2011;181(1):17–22. Epub 2011/05/13. doi: 10.1016/j.vetpar.2011.04.018 . 21561715

[52] Shakya M, Kumar B, Nagar G, de la Fuente J, Ghosh S. Subolesin: a candidate vaccine antigen for the control of cattle tick infestations in Indian situation. Vaccine. 2014;32(28):3488–94. Epub 2014/05/06. doi: 10.1016/j.vaccine.2014.04.053 . 24795229

[53] Blouin EF, Saliki JT, de la Fuente J, Garcia-Garcia JC, Kocan KM. Antibodies to Anaplasma marginale major surface proteins 1a and 1b inhibit infectivity for cultured tick cells. Veterinary parasitology. 2003;111(2–3):247–60. Epub 2003/01/18. doi: 10.1016/s0304-4017(02)00378-3 . 12531299

[54] Munderloh UG, Jauron SD, Fingerle V, Leitritz L, Hayes SF, Hautman JM, et al. Invasion and intracellular development of the human granulocytic ehrlichiosis agent in tick cell culture. J Clin Microbiol. 1999;37(8):2518–24. Epub 1999/07/16. doi: 10.1128/JCM.37.8.2518-2524.1999 10405394 PMC85271

[55] Liu L, Sonenshine DE, Sultana H, Neelakanta G. Identification of a rickettsial endosymbiont in a soft tick Ornithodoros turicata americanus. PLoS One. 2022;17(12):e0278582. Epub 2022/12/07. doi: 10.1371/journal.pone.0278582 36473013 PMC9725135

[56] Taank V, Dutta S, Dasgupta A, Steeves TK, Fish D, Anderson JF, et al. Human rickettsial pathogen modulates arthropod organic anion transporting polypeptide and tryptophan pathway for its survival in ticks. Sci Rep. 2017;7(1):13256. Epub 2017/10/19. doi: 10.1038/s41598-017-13559-x 29038575 PMC5643405

