# Supplementary figures and images for: Anti-tick vaccine candidate subolesin is important for blood feeding and innate immune gene expression in soft ticks
Source: PLoS Negl Trop Dis. 2023 Nov 7;17(11):e0011719. doi: 10.1371/journal.pntd.0011719 (PMC10629623; doi:10.1371/journal.pntd.0011719)

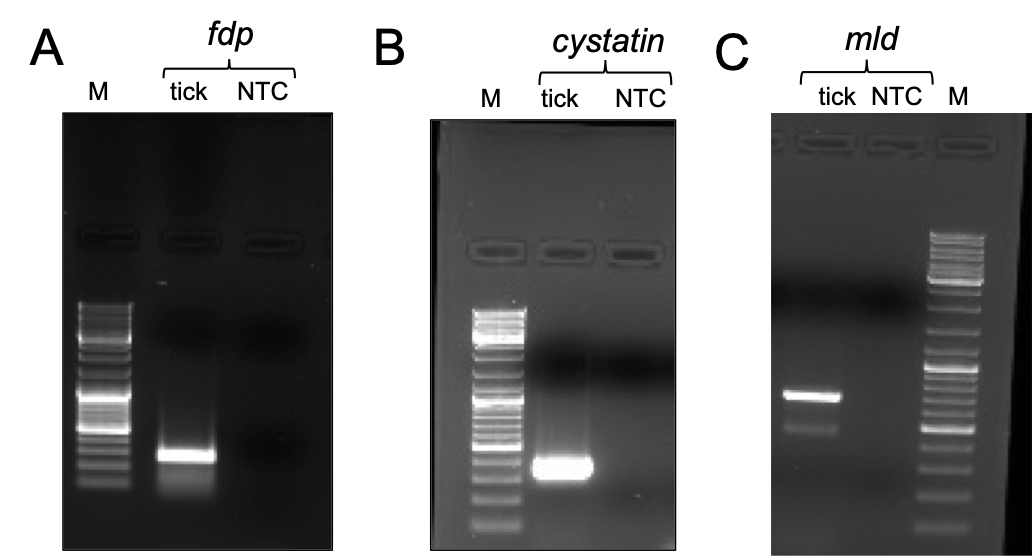

Supplement: S1 Fig — Agarose gel image showing the PCR products of O. turicata americanus fdp (A), cystatin (B), and mld (C) gene fragments from cDNA samples generated from unfed adult female ticks. M indicates marker and NTC indicates no template control. (TIF) [file pntd.0011719.s001.tif]

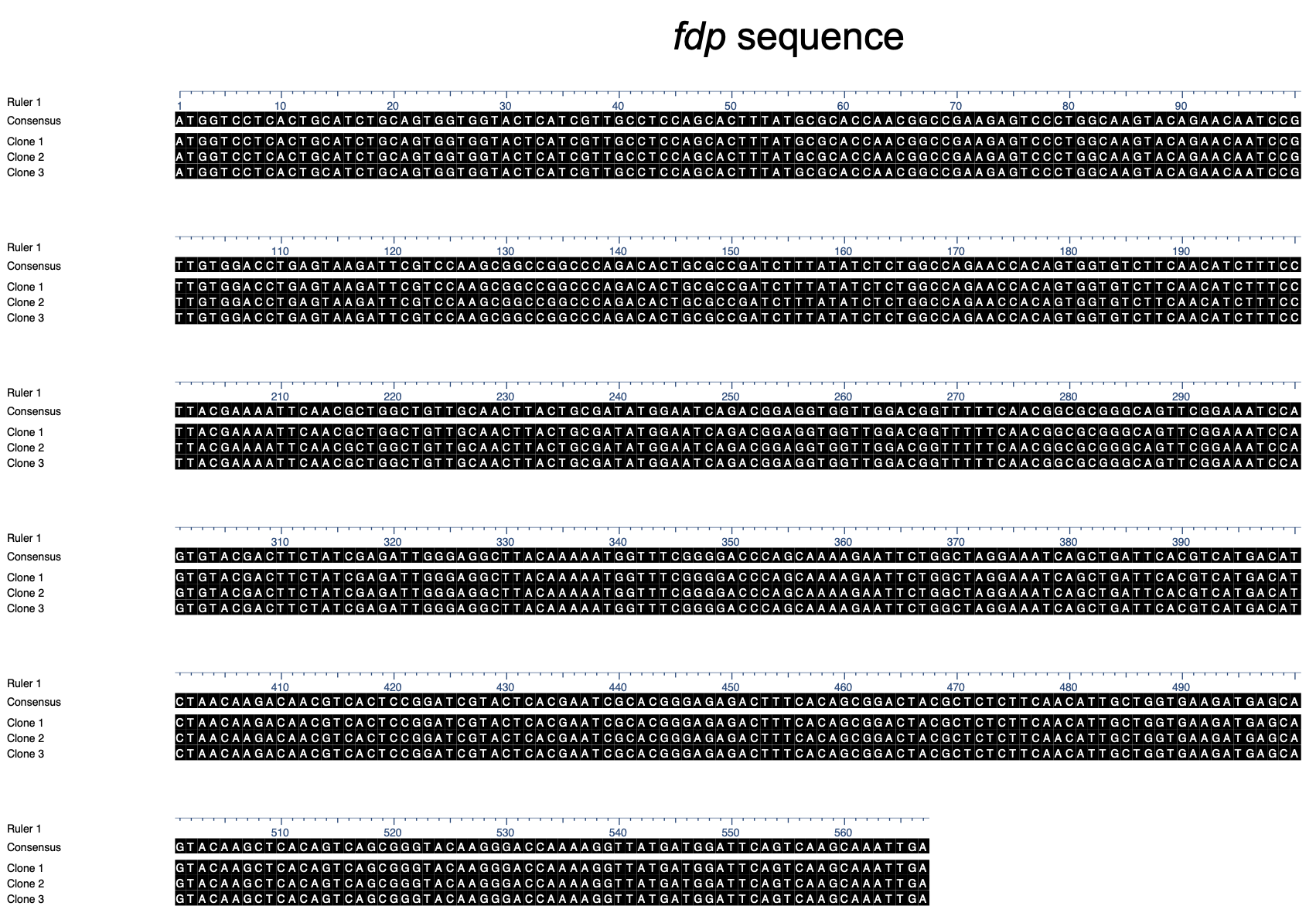

Supplement: S2 Fig — The nucleotide sequence alignment performed using DNA MegAlign software of three clones of O. turicata americanus fdp is shown. Matched sequences are shaded with black color. Consensus sequences are shown below the ruler. Ruler represents nucleobase number. (TIF) [file pntd.0011719.s002.tif]

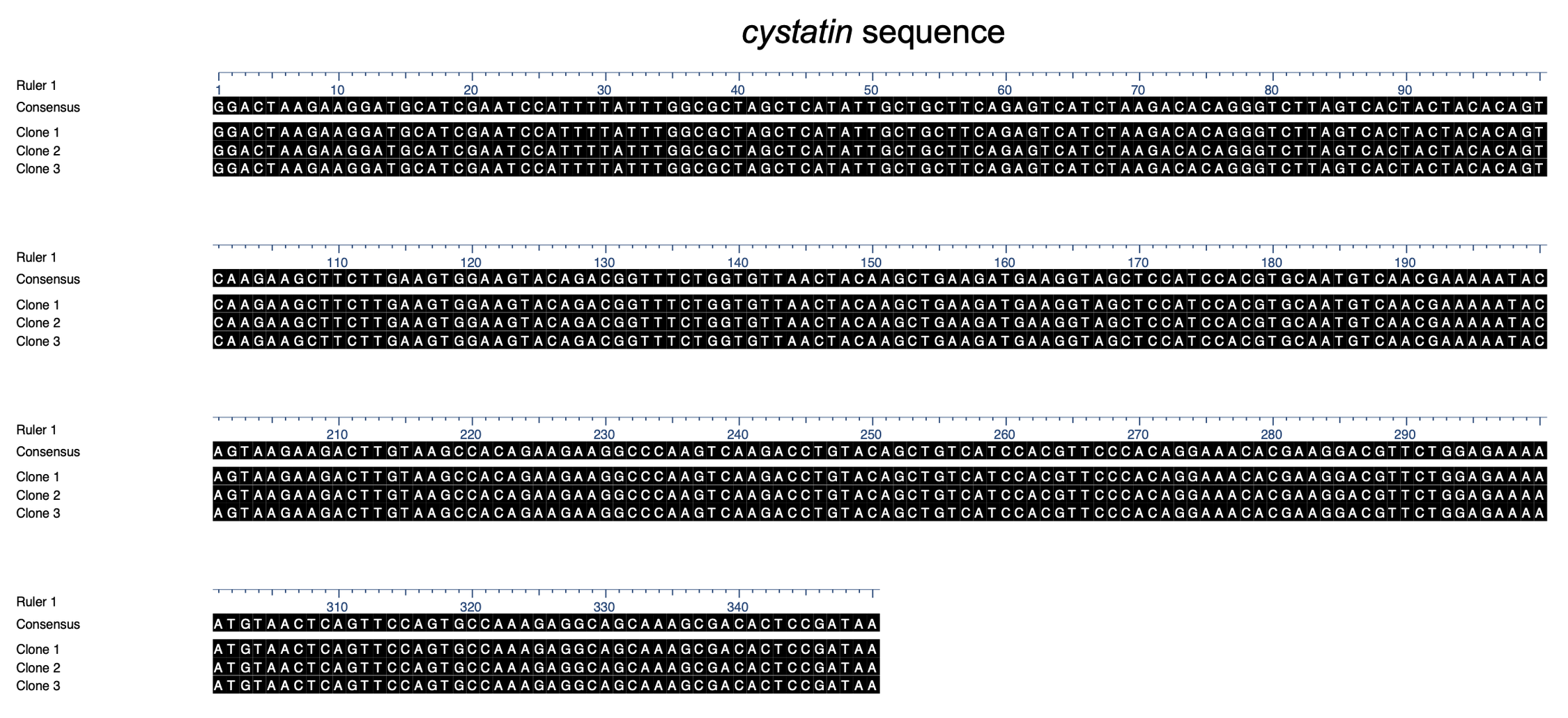

Supplement: S3 Fig — The nucleotide sequence alignment performed using DNA MegAlign software of three clones of O. turicata americanus cystatin is shown. Matched sequences are shaded with black color. Consensus sequences are shown below the ruler. Ruler represents nucleobase number. (TIF) [file pntd.0011719.s003.tif]

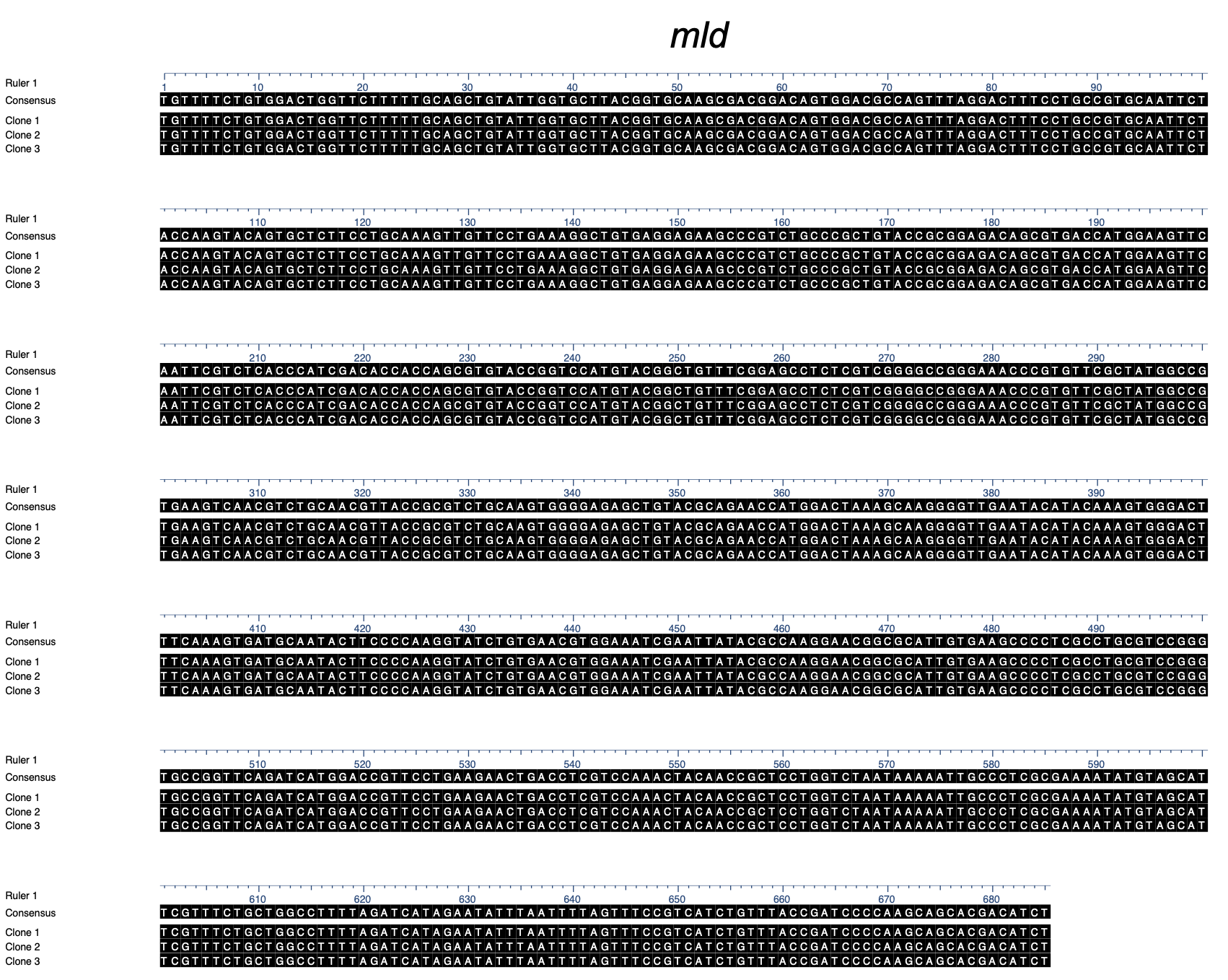

Supplement: S4 Fig — The nucleotide sequence alignment performed using DNA MegAlign software of three clones of O. turicata americanus mld is shown. Matched sequences are shaded with black color. Consensus sequences are shown below the ruler. Ruler represents nucleobase number. (TIF) [file pntd.0011719.s004.tif]

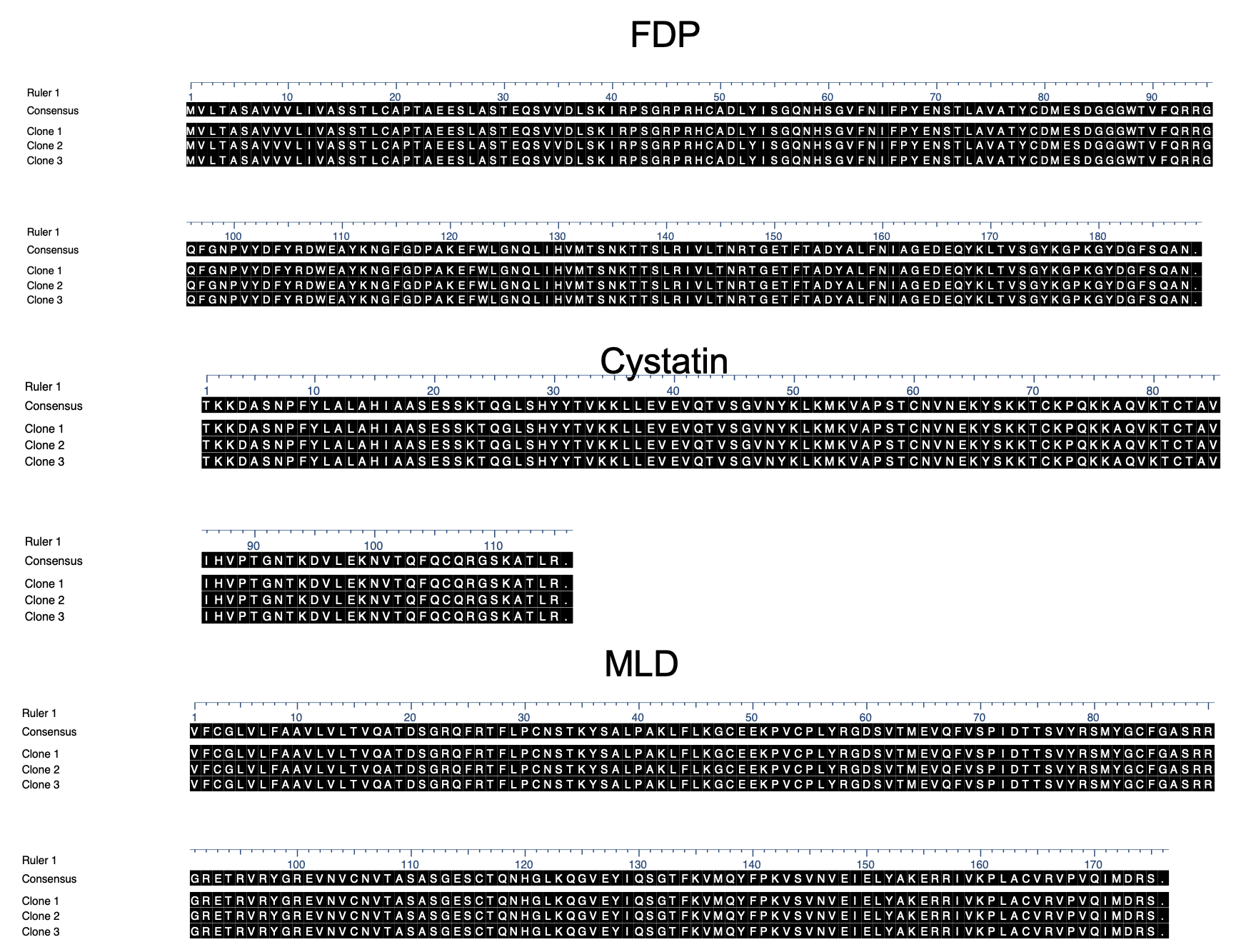

Supplement: S5 Fig — The annotated amino acid sequence alignment performed using DNA MegAlign software of three clones of O. turicata americanus FDP, cystatin and MLD is shown. Matched sequences are shaded with black color. Consensus sequences are shown below the ruler. Ruler represents amino acid number. (TIF) [file pntd.0011719.s005.tif]

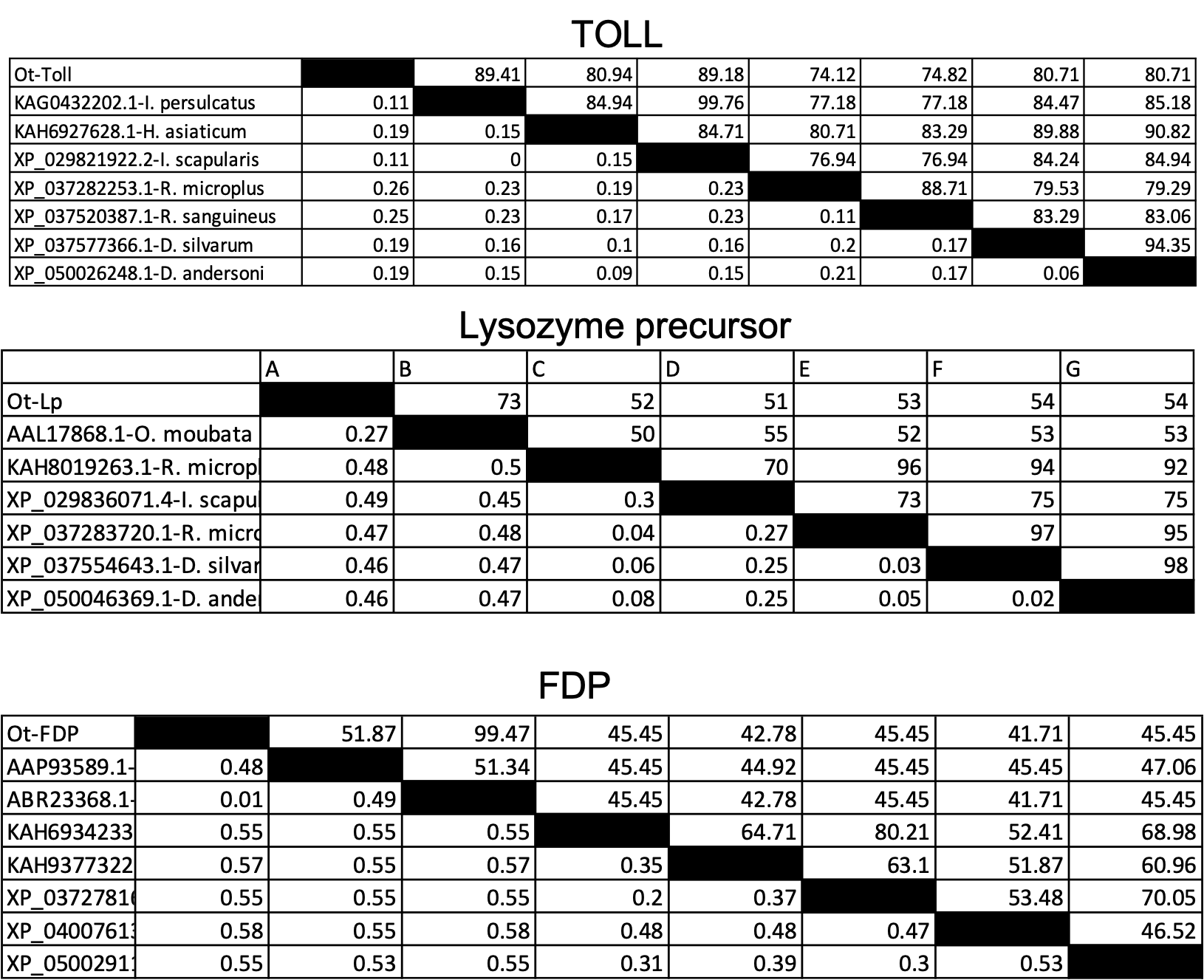

Supplement: S6 Fig — The percent identity (horizontally above black boxed diagonal line) and distance (vertically below black boxed diagonal line) of the O. turicata americanus TOLL, Lp and FDP amino acid sequence in comparison to the ortholog proteins from other hard and soft ticks is shown. The data of percent identity and distance was generated using DNASTAR MegAlign software. GenBank accession numbers and species names are indicated. (TIF) [file pntd.0011719.s006.tif]

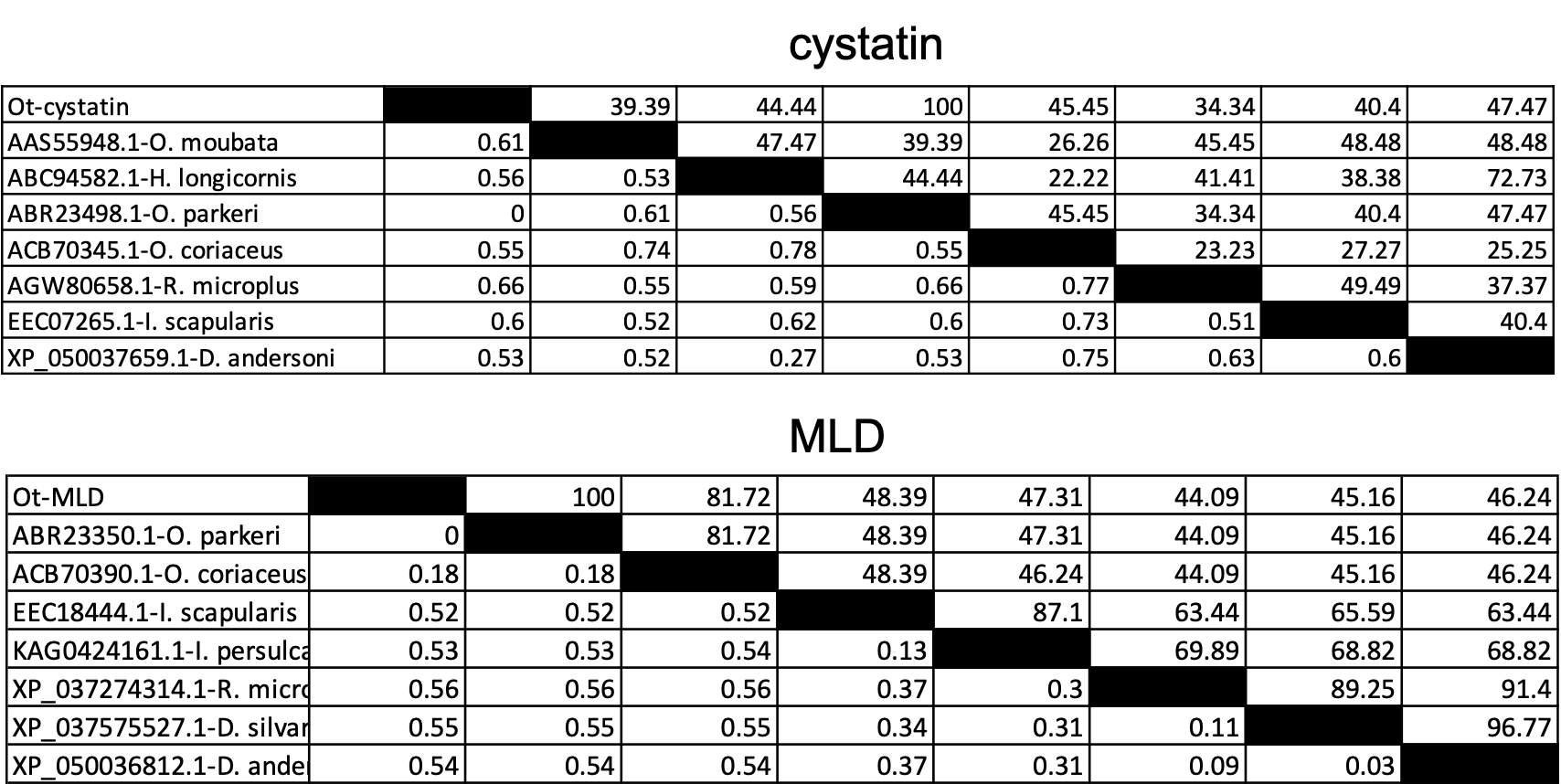

Supplement: S7 Fig — The percent identity (horizontally above black boxed diagonal line) and distance (vertically below black boxed diagonal line) of the O. turicata americanus cystatin and MLD amino acid sequence in comparison to the ortholog proteins from other hard and soft ticks is shown. The data of percent identity and distance was generated using DNASTAR MegAlign software. GenBank accession numbers and species names are indicated. (TIF) [file pntd.0011719.s007.tif]

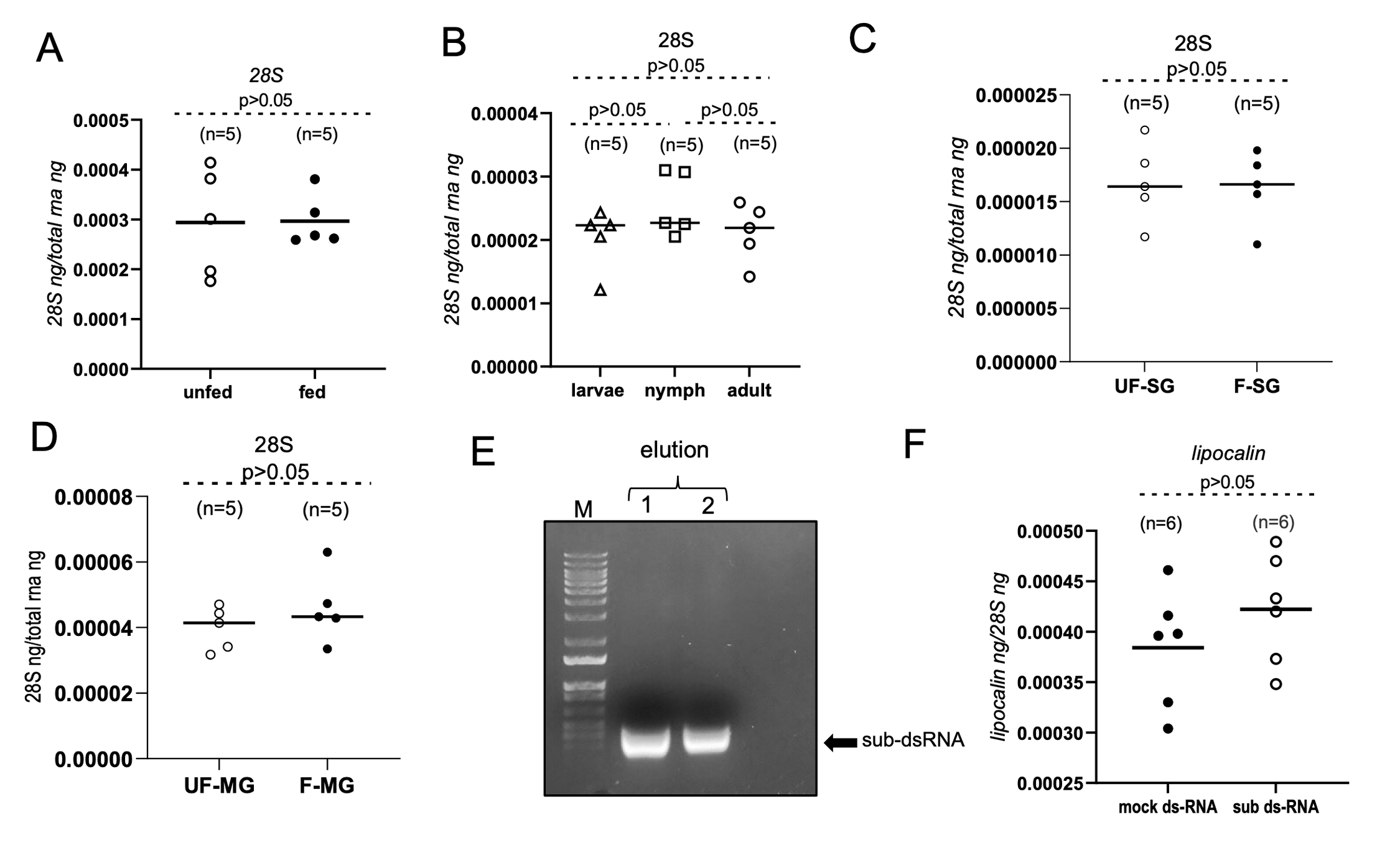

Supplement: S8 Fig — A) QRT-PCR analysis showing levels of O. turicata americanus 28S rRNA transcripts normalized to total RNA in samples generated from unfed and fed adult female ticks (A), unfed larvae, nymphs, and adults (B), salivary glands (C) and midgut (D) isolated from unfed and fed adult female ticks. E) Agarose gel image showing generation and purification of subolesin dsRNA using MegScript RNAi kit is shown. M indicates marker and elution numbers (1,2) are indicated. Arrow indicates sub-dsRNA fragment. F) QRT-PCR analysis showing levels of O. turicata americanus lipocalin gene transcripts normalized to 28S rRNA. In A and C, each circle represents one tick. n indicates number of ticks per sample. P-value from Student’s t-test is shown. (TIF) [file pntd.0011719.s008.tif]

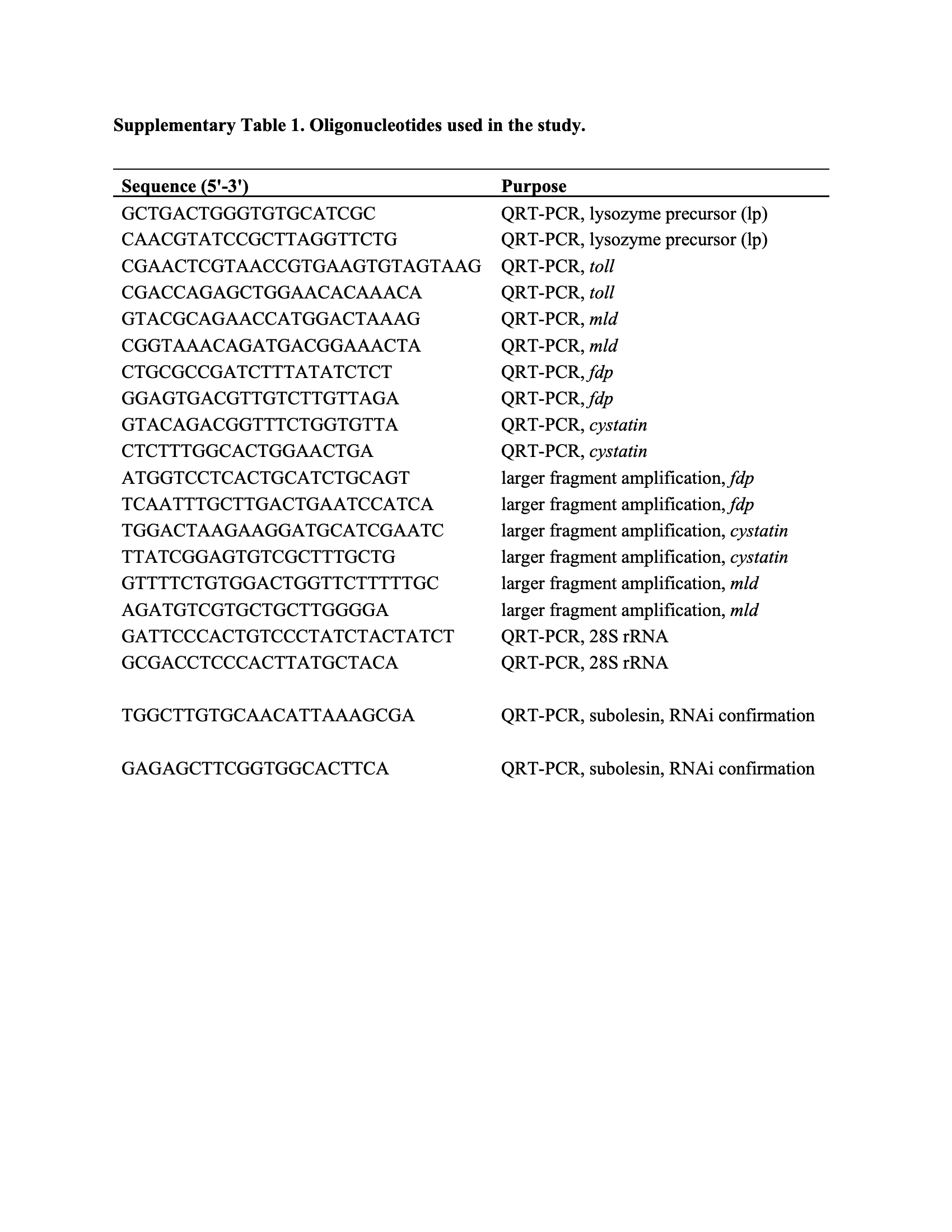

Supplement: S1 Table — Oligonucleotides used in this study are listed in this table. (TIF) [file pntd.0011719.s009.tif]
